# Supplementary material for: The megabiota are disproportionately important for biosphere functioning
Source: Nat Commun. 2020 Feb 4;11:699. doi: 10.1038/s41467-020-14369-y (PMC7000713; doi:10.1038/s41467-020-14369-y)
Supplement: Supplementary file 1 — Supplementary Information [file 41467_2020_14369_MOESM1_ESM.pdf]

The Megabiota are disproportionately important for biosphere functioning

Brian J. Enquist[1,2*], Andrew J. Abraham[3], Mike B. J. Harfoot[4,5], Yadvinder Malhi[6] and Chris Doughty[3]

[1]Department of Ecology and Evolutionary Biology, University of Arizona, AZ 85721, USA.

[2]The Santa Fe Institute, 1399 Hyde Park Rd, Santa Fe, New Mexico 87501, USA.

[3]School of Informatics, Computing, and Cyber Systems, Northern Arizona University, Flagstaff, AZ. 86011, USA

[4]United Nations Environment Programme World Conservation Monitoring Centre, Cambridge, United Kingdom

[5]Computational Science Laboratory, Microsoft Research, Cambridge, United Kingdom

[6]Environmental Change Institute, School of Geography and the Environment, University of Oxford, Oxford, OX1 3QY, UK.

Table of Contents

SUPPLEMENTARY METHODS 4

Applying Metabolic Scaling Theory to the Megabiota 4
Background of Metabolic Scaling Theory 4
Allometric scaling of metabolism. 4
The allometry of biological time. 4
Implications for ecology. 5
Applying Theory to the Megabiota 5
(i) Mortality and extinction risk. 5
(iv) Implications for ecosystem fertility: Nutrient diffusion and nutrient cycling. 12
The Madingley General Ecosystem Model (GEM). 14
Mass-based feeding order. 15

Experimental constraints 16

GEM - Ecosystem Metric Derivation 16

Heterotroph Biomass. 16

Heterotroph Metabolism. 16
Nutrient Diffusivity. 17

SUPPLEMENTARY RESULTS 19

Regional heterotrophic biomass 19

GEM maps of predicted heterotroph metabolism, nutrient diffusivity and autotroph biomass 19
Heterotroph metabolism. 19
Heterotroph nutrient diffusion. 19
Autotroph biomass. 19
Global ecosystem measures summarized by trophic group 19
Nutrient sensitivity tests 20
Spatial variation in abundances and impact of reduction of heterotrophic biomass 20

SUPPLEMENTARY DISCUSSION 21
The potential role of ecological compensation of the smallest plants and animals. 21
The next steps in assessing the role of compensatory processes. 21
An experimental field test of forest compensatory responses to the loss of the megabiota. 21

Implications for ecosystem trophic stability and species diversity. 22

Implications for disease and human health. 23

Caveats 24

Next step research questions in Megabiota research 25

SUPPLEMENTARY TABLES 27
Supplementary Table 1: Total Characteristics of cohorts used in the three experimental simulations. 27
Supplementary Table 2: Total global nutrient diffusivity using three different mass-based scaling coefficients. 27

SUPPLEMENTARY FIGURES 28
Supplementary Figure 1. 29
Supplementary Figure 2 30

Supplementary Figure 3. 31
Supplementary Figure 4. 32
Supplementary Figure 5. 33
Supplementary Figure 6. 34
Supplementary Figure 7. 35
Supplementary Figure 8. 36
Supplementary Figure 9. 37

Supplementary Methods

Applying Metabolic Scaling Theory to the Megabiota

A key component of Metabolic Scaling Theory or MST focuses on the role of organismal size in controlling variation in metabolic rate [1–3] and the role of many other organismal traits that secondarily also influence variation in metabolism and growth [4–7]. Allometry is the study of how changes in organismal size influences biological form and function [8]. Most variation in biological rates and times can be described by differences in body size. For example, variation in the physiological functioning of organisms, the timing of reproductive and growth events, how long organisms live; the turnover and residence time of energy and nutrients in a population, and how long it takes populations to recover and respond to changes in climate all change in predictable ways with the size of an organism [9]. MST proposes a unified theoretical framework to link how variation in body size then influences variation in multiple biological phenomena via allometry how other factors such as climate and ecological interactions influence variation in metabolism [1–3,10–17].

Background of Metabolic Scaling Theory

Allometric scaling of metabolism. A central component of metabolic scaling theory or MST is how various traits of organisms change or scale with changes in their body size. The study of how traits change with body size is called allometry. One of the most studied allometric relationships is how the metabolic rate, B, scales or change with variation in the body mass of the organism, m. Variation in organismal metabolism also drives variation in organismal growth, resource consumption, and overall ecological impact. For both plants and animals, variation in B scales with m as a fractional exponent (approximately 0.75 power), rather than linearly (1.0 power), with body mass, m, where $B \propto m^{0.75}$ (see Refs [18,19]). Exponents less than 1 means that the energetic and resource requirements per unit mass decrease as organismal size increases. For a 0.75 exponent, this means that the resource or metabolic and resource demands per unit body mass or B/m *decreases* with body mass to the fourth root of body mass,

$$B \propto m^{0.75} ; \frac{B}{m} \propto m^{-0.25} \qquad (1)$$

Following the literature [18,19], to start, here we start by utilizing 'quarter-power' scaling exponent values here (3/4, -1/4 etc.). This simplified approach purposefully ignores variation in the reported scaling exponents within and between taxa. As we discuss below, our overall conclusions do not depend on the exact values of these scaling exponent if the scaling exponent for metabolism, $B \propto m^b$ where b is less than 1.0 and that $B/m \propto m^{1-b}$.

The allometry of biological time. Variation in the timing of biological events – what we call biological time, t_b also scales with body size. In general, biological times, t_b, increase with the size of the organism and approximate the fourth root of body mass [20,21] where

$$t_b \propto m^{0.25} \qquad (2)$$

Thus, biological times –reproductive age, longevity, gestation time, physiological response times, life history times, population doubling times, whether at the physiological, population, or ecosystem level, all tend to increase with the largest size of the organism. Thus, larger organisms operate on longer time scales and respond to different cycles of climate variation. The fact that organisms follow a 'time allometry' means that differences in body size will have disproportionate effects that ramify throughout populations, communities, and ecosystems and impact human resource requirements and land use. Also, because B and B/m scale with exponents that differ from 1 and 0

respectively, reductions in the size range of organisms on Earth has profound conservation implications.

Implications for ecology. To meet their metabolic demands and to avoid extinction, natural selection [22–24] has led to larger organisms utilizing more space. Because of the allometry of metabolism, they require more area to obtain and forage for necessary resources and to maintain minimum viable number of individuals. The exclusive foraging area used by an individual of the territory or home range used (A) scales with body size, varying as $A = c\, m^{1.0}$, [25,26] where c is a constant that may or vary across species and is also influenced by trophic-level [25–27]. The difference in the slopes (exponents) of these allometric equations means that the energy requirement of an individual per unit area of its territory decreases with increasing body size,

$$B/A \propto cm^{-0.25} \quad (3)$$

Given Supplementary Equations 1-3, larger organisms retain the nutrients and energy in their vascular and digestive networks (and guts in animals) for a longer period of time. Thus, larger organisms forage for resources over larger areas. They have larger home range sizes or canopy and rooting volumes. Larger animals cover a larger area and travel more widely. Similarly, larger plants have larger canopies and rooting volumes. For animals, longer gut passage times and larger home range requirements means that larger animals transport nutrients processed in their dung and urine over larger areas. Further, the decrease in energy required per unit mass (from above) enables large species to feed on lower quality foods and to include a much wider array of items in the diet [28]. As we discuss below, these scaling relationships underscore the disproportionate metabolic impact of the largest animals and plants but also points to their susceptibility to human land use change, harvesting and hunting, and climate change.

Applying Theory to the Megabiota

(i) Mortality and extinction risk.

The megabiota are disproportionately more sensitive to population declines and extinction given climate change and human land use and resource extraction. This is due to three key factors: (1) the mortality risk in extreme events, R; (2) the scaling of population time to recovery and per capita fecundity rate, F; and (3) the minimum area needed for persistence, A_m.

Mortality risk in extreme events, R. First, some groups of larger organisms are often closer to operating at biophysical and abiotic limits. This appears to be the case for plants and many aquatic organisms. For these organisms, climate change will differentially emphasize these limits in the largest individuals exposing them to increased risk of mortality. Further, larger and taller trees and some animals are also closer to biomechanical and hydraulic height limits [29,30] and more prone to hydraulic and mechanical failure. We give two examples.

As a first example, larger trees are closer to hydraulic limits so are more prone to drought and heat stress[31–33]. Indeed, the basic tenet of the 'hydraulic limitation hypothesis' is that taller trees exhibit increased stomatal closure due to an increase in hydraulic resistance with tree height combined with the need to maintain a minimum leaf water potential to avoid catastrophic embolism [34]. Metabolic Scaling Theory applied to plant hydraulics [13] predicts that, for realistic size ranges across plants, the resistance per path length will increase significantly as the water transport distance within the plant increases. As a result, MST applied to understanding the linkages between climate and maximum plant size [35] shows that hotter and more dry climates will lead to a reduction in maximum tree size [36]. For example, the risk of tree mortality, R, due to drought is size dependent, killing the largest trees [33]. Drought mortality occurs when the available supply of water to a plant cannot meet the evaporative demand for water from the plant [31]. McDowell et al. link variation in maximum size (via maximum

plant height, h_{max}) to variation in climate by utilizing a hydraulic corollary of Darcy's Law [31] where the maximum height (size) a tree can reach is given by

$$R \propto h_{max} \propto \frac{A_S k_S[\psi_S - \psi_l]}{G\eta A_l V};\ R \propto h_{max} \propto V^{-1} \qquad (4)$$

where G is the canopy-scale water conductance (mol m^{-2} s^{-1}), h_{max} is the maximum plant height that can be hydraulically supported (m), As is the conducting area (cm^2), A_l is the leaf area (m^2), k_s is the specific conductivity (m s^{-1}), η is the water viscosity (Pa s), $\psi_S - \psi_l$ is the soil-to-leaf water potential difference (MPa) and V is the vapor pressure deficit (kPa) of the atmosphere. Increases in temperature from climate change and increases in drought conditions increase the vapor pressure deficit, V, leading to a reduction in h_{max}. Supplementary Eqn 4 predicts that the size limit in vegetation is constrained by the environment – specifically the vapor pressure deficit, V. With climate change, future droughts will likely be hotter and drier increasing V. Supplementary Eqn 4 predicts that $h_{max} \propto V^{-1}$ which indicates a predicted decrease in h_{max} (the largest trees). As $h_{max} \propto m_{max}^{3/8}$ then in the case of trees, the mortality risk in extreme events $R \propto m_{max}^{3/8}$.

As a second example, changes in climate also influence the body size ranges in aquatic organisms. A change in temperature, oxygen content and aquatic biochemistry directly affect the ecophysiology of marine water-breathing organisms [37,38]. In aquatic habitats climate changes induces changes in water temperature, oxygen, and pH. These changes select against large body sizes leading to a reduction of body size of marine fishes. The maximum body mass of marine fishes and invertebrates is fundamentally limited by the balance between energy demand and supply, where energy demand is equal to the energy supply [39]. Change in oxygen levels in aquatic environments can change the capacity for growth in aquatic water-breathing ectotherms. Oxygen-limitation is one of the fundamental mechanisms determining biological responses of fish to environmental changes, from cellular to organismal levels. With climate change, oceans and freshwater habitats will be characterized by warmer temperatures, decreased pH, and reduced oxygen. As a result, future climates will have a more detrimental impact on the growth and mortality of larger fish leading to reductions in body sizes and potentially exacerbating feedbacks to climate change [38].

Scaling of population time to recovery and per capita fecundity rate, F. Second, large animal and plant populations have lower rates of fecundity than smaller animals[20,40]. The per capita fecundity rate, defined as F, or the offspring mass produced per population biomass per unit time ($g \cdot g^{-1} \cdot t^{-1}$), scales inversely with body size as $F \propto m^{-0.25}$. Further, larger animals or plants also require more time to grow and reach reproductive maturity do that generation times increase with increases in size $t_b \propto m^{0.25}$. As we show below, given the allometry of F and tb , in times of heighted risk of mortality to the largest individuals, extinction risk will then be inversely proportional to organismal body size. This is because larger organisms have slower demographic rates (t_b) and their populations take longer to recover from environmental perturbations, disturbances, harvesting, and hunting [41].

Minimum area needed for persistence, A_m. Third, larger organisms are more constrained to have larger geographic ranges than smaller body sized organisms[42]. In order to maintain viable global population sizes above a critical threshold to avoid stochastic extinction, large body sized animals are more constrained to have larger geographic range sizes[24]. This is because larger organisms tend to be more locally rare[43,44] but have larger home range sizes, A.

Thus, the minimum area required for persistence (A_m) as: $A_m \propto N_m A$ where A is home range size and N_m is the minimum number of individuals required to avoid extinction in the absence of immigration[22,23]. These equations set the boundary for persistence and apply to the largest species able to persist in each area or landmass in the face of extinction. The minimum number of

individuals required to avoid stochastic extinction N_m is thought to be approximately independent of body size [22,23]. Thus, globally, a minimum global number of individuals is necessary in order to avoid stochastic extinction [24]. Given that $A \propto m^1$ we have $A_m \propto m^0 m^1$ indicating that the minimum area required for persistence (minimum geographic range size) to avoid stochastic extinction must increase with body size.

This constraint on A_m is likely more pronounced in animals where population densities tend to be much lower than in plants [45]. Larger body sized organisms tend to have larger geographic ranges [22,23], but there is a downside to have a larger geographic range size. Large range size increase the likelihood that a species will encounter humans and human modified environments. As a result, for any given area (e.g. an island or a reserve or habitat remnant) larger organisms are more at risk of stochastic events or regime changes (e.g. increase in hunting pressures) and will have smaller population sizes. As the probability of extinction increases with smaller population size [46,47] the probability of extinction is strongly influenced by total population size and hence the size of the geographic range. Indeed, recent analyses show that during times of rapid climate change and human colonization of the major continents extinction has differentially eliminated the largest species with the smallest geographic ranges [48,49] (see Figure 1 in main text).

Next, we show how in times of rapid change, the scaling of *R*, *F*, and A_m with body size can combine to influence species extinction risk E_λ. Specifically, species extinction risk, E_λ, is a function of the risk of mortality due to extreme events, *R*; the per capita fecundity rate, *F*; and the size of the geographic range, *A* (a proxy for the total population size). Given the above arguments, we can express the risk of extinction, E_λ during periods of increased land use change, hunting and exploitation, and climate change, as a function of body mass, *M*. First, the hypothesized positive relationship between mortality risk in extreme events, *R* (see Supplementary Eqn 4) would predict that *E* scales positively with body size. Second, given the negative relationship between the per capita fecundity rate per unit time, *F* and body mass (see Supplementary Eqn 2) predicts that in times of rapid habitat decline, hunting and exploitation, and climate change, the decreased per capita fecundity rate characteristic of larger body sizes leads to increased risk of population declines and thus extinction rates. Lastly, given the above, large body sized animals are more constrained to have larger geographic range sizes [24]. Thus, the minimum area required for persistence (A_m) there is large ranged species are and a negative function of the size of the geographic range, *G*. Each variable, *R, F, A* are functions of body size, *M* where

$$E_\lambda \propto f[R(m^b) \cdot 1/F\,(m^{-c}) \cdot A_m(m^d)] \propto m^{b+c+d} \qquad (5)$$

Here, variation in the risk of extinction, *E* is governed by variation in body mass and the exponents *b, c,* and *d*, can in principle vary with the type of organism and environment. For example, the mortality risk scaling exponent, *b*, due to climate change related events in mammals may be close to zero [50] so that $R(m^b) \approx 1$. In contrast, in plants and aquatic animals $b > 0$ (see Eq. S4), so $E \propto R \propto m^b$. Across most organisms, the fecundity per unit time, *F*, is inversely proportional to body size so $E_\lambda \propto 1/F$ where $F \propto m^{-c}$ where the value of *c* approximates 1/4 [9].

From above, the risk of extinction due to reductions in range size is expected to scale in proportion with geographic range size $E \propto G \propto m^d$ where the value of *d* is likely positive [48]. With a reduction in geographic range size, due to land use or climate change it follows that extinction probability should be positively corrected directly with body size [51]. As an approximation, if $b \sim 1$, $d \sim 1$, and $c \sim 0.25$ then we would expect that $E_\lambda \propto m^{2.5}$ (see also [22,23]). However, we expect that depending on the organism and environmental driver that the values of *b,d,* and *c* will likely vary. Thus, Supplementary Eqn 5 indicates that E_λ will increase with *m* but the magnitude of extinction risk will

depend on the values of the exponents b ,*d*, and *c*. Therefore, compared to smaller body sized flora and fauna, in a time of rapid climate change and reductions of geographic range in the largest individuals Supplementary Eqn 5 predicts that increasingly larger body sized species face increased risk of extinction [48,52].

The probability of a species being threatened by extinction does indeed increase with body size in birds, mammals, and cartilaginous fishes[53]. Ripple et al. found that an order-of-magnitude increase in body mass was associated with estimated increases in the odds of being threatened of 294% for large bony fishes, 184% for large amphibians/ reptiles, 107% for birds, 92% for cartilaginous fishes, 67% for mammals, and 27% for large vertebrates pooled [53]. But this study also found that for some groups there was evidence that smaller body sized species in bony fish and amphibians and reptiles also had increased extinction risk indicating that the relationship with E_λ and m is more complicated in some groups. Such complex dynamics may reflect differing functions for relating $R(m), F(m),$ and $A_m(m)$ to E_λ.

(ii) Ecosystem scaling: Stocks and total biomass.

Increases in the largest body sizes and the area devoted to the macrobiota disproportionately impact ecosystem fluxes and standing stocks of carbon, nitrogen, phosphorus and other elements. Metabolic scaling theory provides a theoretical foundation to predict how changes in the macrobiota will influence ecosystem and trophic stocks, M_{Tot}, and fluxes, J_{Tot}. Specifically, ecosystem scaling relationships are the result of three important inputs – the distribution of body sizes (the size spectra), f, the allometric relationship between body size and metabolism, B (the allometric scaling of metabolism), and the allometric relationship between the primary measure of body size, x (e.g. stem radius, r, height, h, or mass, m) and body mass (the allometric scaling of body dimensions).

For a given trophic level, the total metabolism or resource flux (utilization rate) or J_{tot} (kg yr^{-1}) of a given resource i, such as nitrogen, water, carbon etc, is influenced by two functions, the size distribution, f and the allometry of of metabolism, *B*. Similarly, for a given trophic level, the total standing stock such as the standing biomass, M_{Tot} is influenced by two functions, the size distribution, f and the allometry relating the primary measure of body size, *x*, to body mass, *m*. Both can be expressed as functions of organismal size or mass, *m*. This can be written as

$$J_{Tot} = J \cdot A = \int_{m_{min}}^{m_{max}} \tau \kappa_i^{-1} B(x) f(x) dm \; ; \; M_{Tot} = M \cdot A = \int_{r_0}^{r_{max}} m(x) f(x) dr \qquad (6)$$

where A (m^2) is the geographic area under consideration, J (kg m^{-2} yr^{-1}) is the resource flux per unit stand area, τ (s yr^{-1}) is time unit conversion factor, $\kappa_i = B/\dot{R}_i$ (J kg^{-1}) is the resource-to-energy conversion factor of resource i, and $\dot{R}_i$ (kg s^{-1}) is the supply rate of resource i [54].

As we discuss below, if the size range is large so that the size of the smallest individual, m_{min} is much smaller than the size of the largest individual, m_{max} so that $m_{max} >> m_{min}$, then the scaling of J_{tot} can be expressed in terms of the size of the largest individual, m_{max}. Further, the scaling of ecosystem fluxes, J_{tot} as well as ecosystem standing stocks of biomass or nutrients can also be shown to scale with the total biomass, M_{Tot}. These ecosystem scaling relationships depend on two specific functions: *B(m),* the allometry of metabolism, *f(m),* the size distribution or size spectra, and what the primary measure of body size, x, is in the system of interest.

Scaling of ecosystem standing stocks of biomass: Plants. The total biomass within a given trophic level M_{Tot} (kg) is influenced by the size distribution, f and the allometry of organismal form. In the case of autotrophs, in this example forests, the total phytomass can be related by the primary size measure as defined by West et al. (2009)[10,55] – stem radius of a tree, r, where given the size distribution function, f(r) representing the frequency of individuals per unit stem radius, c_n, where $f(r) = dn/dr = c_n r^{-2}$ within units of m^{-1} and the allometric function between stem diameter and biomass, $m(r) = (r/c_m)^{8/3}$ with units of cm $kg^{-3/8}$

$$M_{Tot} = M \cdot A = \int_{r_0}^{r_{max}} m(r)f(r)dr$$
$$= \int_{r_0}^{r_{max}} \left(\frac{r}{c_m}\right)^{\frac{8}{3}} (c_n r^{-2})dr$$
$$= \frac{c_n}{c_m^{8/3}} \int_{r_0}^{r_{max}} r^{2/3} dr$$
$$= \left(\frac{3}{5}\frac{c_n}{c_m^{8/3}}\right)[r_{max} - r_0]^{5/3}$$
$$M_{Tot} \approx \left(\frac{3}{5}\frac{c_n}{c_m^{8/3}}\right) r_{max}^{5/3} \qquad (7)$$

Where *A* (m^2) is the geographic area under consideration, r_{max} is the size or radius of the largest plant stem in the sampled community and r_0 is the size of the smallest individual. If the size of the largest individual $r_{max} >> r_0$ then the total stand biomass, M_{Tot} should scale approximately with the size of the largest individuals. Specifically, M_{Tot} is predicted to increase as the 5/3 power (1.67) of the largest stem radius (Fig. 3). This relationship can also be written in terms of the mass of the largest tree or individual m_{max} (kg) by substituting $r_{max} = \frac{1}{c_m} m_{max}^{3/8}$, gives

$$M_{Tot} \approx \left(\frac{3}{5}\frac{c_n}{c_m^{8/3}}\right)\left(\frac{1}{c_m} m_{max}^{3/8}\right)^{5/3}$$

$$M_{Tot} \approx \left(\frac{3}{5}\frac{c_n}{c_m^{8/3} c_m^{5/3}}\right) m_{max}^{5/8} \qquad (8)$$

Thus, MST predicts that as the size of the largest tree increases the total standing biomass and carbon content of a forest increases disproportionately to the size of the largest individual.

Scaling of ecosystem standing stocks of biomass: Animals. Similarly, for animals, using the method in Supplementary Eqn 6 and 7, the standing stock of animal biomass in a given trophic level can be related to the size of the largest animal in the community or ecosystem. Here, we start with assuming that the critical size dimension is mass, instead of stem diameter or radius as is the case with terrestrial plants (see West et al. 2009; Enquist et al. 2009). As such, the starting size distribution is f(m) where
$N = c_a m^{-\frac{3}{4}}$. The total biomass of all animals, M_{Tot} is given as

$$M_{Tot} = M \cdot A = \int m(m)f(m)\, dm = \int m \cdot c_a m^{-\frac{3}{4}}\, dm$$
$$= c_a \int m^{\frac{1}{4}}\, dm \approx \frac{4}{5} c_a\, m_{max}^{\frac{5}{4}} \qquad (9)$$

Where again A (m^2) is the geographic area under consideration, c_a is the frequency of individuals per unit organism mass, m, m_{max} is the mass of the largest animal, and c is a constant from integration. As a result, Supplementary Eqn 9 predicts a super linear scaling of animal biomass with the size of the largest animal. Specifically, Supplementary Eqn 9 predicts that total trophic level biomass will increase to the 5/4 or 1.25 power of the size of the largest individual. Like Supplementary Eqn 8 for plants, Supplementary Eqn 9 predicts that ecosystems with larger animals will contain more biomass than in ecosystems where the largest animals have been lost. However, unlike plants, because of the superlinear scaling (exponent greater than 1) the total animal biomass is more sensitive to losses of the largest animals.

Assessing predictions. We tested the above predictions using data on forest biomass and maximum forest tree size from Stegen et al. (2011). Stegen et al. [56] utilized a consistent sampling regime based on 0.1-ha forest inventory plots (based on the methods of Phillips & Miller [57]). The data stem from 267 forest plots distributed across the Americas from 40.7° S to 54.6° N latitude. Plots known to be in early successional forests were excluded from the dataset. Within each plot, consisting of 10 transects with dimensions 2 m x 50 m, all woody stems greater than or equal to 2.5 cm diameter at breast height (including lianas) were measured and identified to species or morphospecies. Biomass of each plot was estimated using equations specific to tropical forest type, liana and temperate angiosperm and gymnosperms. The best single predictor of variation in forest biomass is the size of the largest tree in that forest (Fig. 3). The fitted slope of the relationship (the scaling exponent) is 0.62, which is indistinguishable from the predicted scaling function from metabolic scaling theory where the total biomass should scale as maximum tree size to the 5/8 or 0.625 power.

Detailed tests for animals are needed. Our Madingley simulations are among the first assessments of how the loss of the megabiota can influence ecosystem and biosphere functioning. These simulations provide tentative support for the prediction (Supplementary Eqn 9) that a reduction in the maximum size, m_{Tot} of animal reduces the total heterotrophic biomass (Fig. 5D).

(iii) Implications for scaling of ecosystem fluxes

Scaling of total energy, carbon, nutrient pools and resource fluxes: Plants . For plants, we can express the total metabolic rate of all individuals B_{tot} as a function of the radius of the size of the largest tree, r_{max}

$$\begin{aligned} B_{Tot} &= B \cdot A \\ &= \int B(r) f(r) dr \\ &= b_0 c_n \int_{r_0}^{r_{max}} dr \\ &= b_0 c_n [r_{max} - r_0] \\ &\approx b_0 c_n r_{max} \end{aligned} \tag{10}$$

where A (m^2) is the stand area under consideration B (W m^{-2} yr^{-1}) is the metabolic flux per unit stand area, and $B(r) = b_0 r^2$ (W) is the whole-plant metabolic rate.

This framework can also be extended to carbon and resource fluxes. For example, the total stand resource utilization rate J_{tot} (kg yr^{-1}) of a given resource i , such as nitrogen, water, carbon etc, can be written as

$$
\begin{aligned}
J_{Tot} &= J \cdot A \\
&= \int_{r_0}^{r_{max}} \tau \kappa_i^{-1} B(r) f(r) dr \\
&= \int_{r_0}^{r_{max}} \tau \kappa_i^{-1} b_0 c_n dr \\
&= \tau \kappa_i^{-1} B_0 c_n [r_{max} - r_{min}] \\
&\approx (\tau \kappa_i^{-1} B_0 c_n) r_{max}
\end{aligned}
\qquad (11)
$$

where J (kg m^{-2} yr^{-1}) is the resource flux per unit stand area, τ (s yr^{-1}) is time unit conversion factor, $\kappa_i = B/\dot{R}_i$ (J kg^{-1}) is the resource-to-energy conversion factor of resource i, and R_i (kg s^{-1}) is the supply rate of resource i. In terms of fruit, seed production and total absorption of carbon – larger trees will increase in size more, grow more, produce more, and store more.

The disproportionate impact of the largest organisms ramifies to the total productivity of the forest and flux of carbon, water, nutrients. In terms of the total biomass of the forest M_{Tot}, the total energy flux through the entire forest B_{Tot} and the total productivity of the forest GPP and total flux J, we have $J_{Tot} \propto NPP_{Tot} \propto B_{Tot}$.

We can use the above formalization to then relate how variation in maximum plant size then influences total stand biomass. Given, from Supplementary Eqn 7, the predicted scaling relationship between M_{Tot} and r_{max} $where\ M_{Tot} \approx \left(\frac{3}{5}\frac{c_n}{c_m^{8/3}}\right) r_{max}^{5/3}$ we can simplify and define $\eta \equiv \frac{3}{5}\frac{c_n}{c_m^{8/3}}$. Then, from above, we can substitute and relate the total stand biomass to the size of the largest individual where $M_{Tot} \approx \eta\, r_{max}^{5/3}$, $r_{max} \approx \left(\frac{M_{Tot}}{\eta}\right)^{3/5}$ so that we have,

$$
NPP_{Tot} \propto B_{Tot} \approx b_0 \frac{c_n}{\eta^{3/5}} [M_{Tot}]^{3/5} \qquad (12)
$$

Thus, Supplementary Eqn 12 predicts that as the size of the largest individual increases, r_{max} or m_{max}, the total amount of biomass, M_{Tot}, in the forest increases (see Supplementary Eqn 9) and as a result, Supplementary Eqn 12 predicts that increases in M_{Tot} will then result in increased total ecosystem metabolism, B_{Tot} and productivity NPP_{Tot}. The amount of resources (carbon, water, nutrients) that pass through the forest will also increase with increases in the size of the largest individual. As a result, forests with larger trees will increase the total amount of carbon stored in the forest and will produce more biomass. As a rule of thumb, doubling the size of the largest tree in the forest will result in a 5/8th or 0.625 proportional increase in the total forest carbon and a 3/5th or 0.6 proportional increase in carbon, water, and nutrient flux.

Scaling of total energy, carbon, nutrient pools and resource fluxes: Animals: Similarly, for animals, the total resource utilization rate J_{tot} (kg yr^{-1}) of a given resource i, such as nitrogen, water, carbon etc, is proportional to the total metabolism of all animals, B_{Tot}, so that we have

$$
B_{Tot} \propto J_{Tot} = \int_{min}^{max} \tau \kappa_i^{-1} B(m) f(m) dm \approx (\tau \kappa_i^{-1} b_0 c_n) m_{max} \qquad (13)
$$

indicating that just as in with plants increases in the maximum body size of an animals would also increase the total amount of flux through the heterotrophic food web.

As we discuss below (see section on 'The potential role of ecological compensation of the smallest plants and animals for the loss of the largest plants and animals') these predictions can be modified by compensatory dynamics.

Assessing predictions. For plants, as discussed in the main text (see discussion around Fig. 3), several recent studies have assessed the prediction (Supplementary Eqn 12). Forests with larger individuals and more biomass tend to be more productive. Together, recent papers by Michaletz et al. [58,59] Enquist et al.[60,61] and Símova et al.[62] largely support the predictions. Recent insights on the analysis of tree growth have shown that forests with older, larger trees hold more carbon and are more productive (increased total NPP, GPP) than more disturbed secondary forests without large trees [10,54,56,63]. Several recent studies assessing variation in forest support these predictions indicating that NPP and GPP scales with total autotrophic (plant) biomass with exponents indistinguishable from the 3/5th power [59,61,64]. Further, based on theoretical predictions from MST with an increase in tree size there is a disproportionate increase in the size and magnitude of ecosystem fluxes and pools [10,62]. The total energy, carbon, and resource fluxes that characterize vegetation are all directly proportional to the size of the largest individual, meaning that a doubling of stem radius will double the flux [10,54]. Further, the energy, carbon, and resource fluxes are approximately proportional to the largest individual as measured by stem radius [63].

For animals, analysis of data from Brown and Maurer 1989 [65] provides tentative support for a positive relationship between total animal metabolism within and maximum body size. In their dataset, they assessed the relationship between the body mass of a bird species and an estimate of the total energy use of all individuals within that species. Metabolic Scaling Theory predicts that applied to the species-level scale, larger sized species should flux more energy than smaller sized species. The predicted slope of this relationship is $J_{Tot} \propto m_{max}^{5/4}$ (5/4=1.25) does appear to approximate the original relationship observed by Brown and Maurer. This relationship support the predictions that larger body mass animals collectively flux more energy and resources than smaller body sized animals. Further, our Madingley simulations provide tentative support for the prediction (Supplementary Eqn 13 and 14) that a reduction in the maximum size, m_{Tot} of animal reduces the total heterotrophic biomass (Fig. 5D).

(iv) Implications for ecosystem fertility: Nutrient diffusion and nutrient cycling.

The rate at which nutrients and energy diffuse by herbivores across a landscape, ϕ, was empirically derived using a large mammal database. The scaling exponent was 1.17 based on the equation below:

$$\phi = (1-\varepsilon)B\left(\frac{N}{\alpha M_{Tot}^{Plant}}\right)\frac{(A \cdot t_{gut})^2}{2 \cdot t_{gut}} = 0.050\, m_{Herbivore}^{1.17} \qquad (14)$$

Where N is population density (#/km^2), B is metabolic rate (kg dry matter (DM)/#/day), A is day range (km), and t_{gut} is passage time (days). The term αM_{Tot}^{Plant} is an edible autotrophic biomass term and ε is the fraction incorporated into the body. Wolf *et al* (2013) used empirical data to find the allometric exponent for the nutrient diffusion capacity, $\Phi_{excreta} = 0.050 * m^{1.17}$, which is a statistical fit to primary data (i.e. we have complete data for all species but a lower sample size) and our best estimate. The best fit using scaling coefficients from individual parameters (i.e. data are compiled individually compiled together) is 0.053 $M^{1.011}$.

Supplementary Equation14 makes several predictions. If M_{Tot}^{Plant} is approximately constant, then nutrient diffusion across the landscape will scale positively with the mass of an herbivore raised to the κth exponent. Reductions in plant biomass will also decrease nutrient diffusivity. Doughty et al. estimate the scaling exponent for $\phi \propto m_{Herbivore}^{\kappa}$ is $\kappa \sim 1.17$ based on a synthesis of empirical studies. Increases in herbivore size then lead to an increase in nutrient spread across ecosystems and hence boosting the fertility of ecosystems. Thus, increases in herbivore size then lead to an increase in nutrient transport and spread across ecosystems.

Assessing predictions. For animals, we assessed the prediction of Supplementary Eqn 14 that a reduction in the largest sized animal will reduce ecosystem fertility. We took two different approaches:

First, using the same simulation model in Doughty et al 2013; [66] we modeled how reductions in the maximum sized herbivore would then impact that fertility of the soils of the Amazon Basin. Following their exact methodology [66] we calculated the steady state estimate of soil P concentrations in the Amazon basin *prior* to the megafaunal extinctions. The extinctions of the megafauna in South America has led to drastic changes in animal size distributions with 70% of mammal species greater than 10kg going extinct (62 species) since the Pleistocene including such large iconic species as gomphotheres, giant sloths and glyptodonts. With the extinction of large mammals and a continued forecasted reduction in mammal body size, the percentage of the original steady state P concentrations in the Amazon Basin will decrease (Figure 4). This simulation is characterized by lateral diffusivity of nutrients (Φ) by mammals away from the Amazon river floodplain source. In the simulation, animal nutrient transport are modelled via diffusivity of nutrients via ingestion, transport, and eventual defecation. These simulations yield a $\Phi_{excreta}$ value of 4.4 $km^2\ yr^{-1}$. In our simulation, with the extinction of large mammals and a continued forecasted reduction in mammal body size, the percentage of original steady state P concentrations in the Amazon Basin will decrease. As shown in Fig. 4, under a series of size thresholds for the extinct megafauna, we expect a 20-40% reduction in soil steady state P concentrations. For instance, a 5000 kg size threshold removes all animals above 5000 kg and continental P concentrations are reduced by ~10%.

Second our Madingley simulations provide tentative support for the prediction (Supplementary Eqn 14) that a reduction in the maximum size, m_{Tot} of animal reduces total ecosystem fertility (Fig. 5F).

(v) The multiplicative importance of conserving the megabiota and increasing the total area protected

An important implication of the above analytical theory is that the megabiota are also disproportionately more impactful for conservation efforts prioritizing ecosystem functioning. Because the total trophic biomass or the forest biomass will be directly proportional to the land area, A, that is forested [67] doubling the area, A, available for forests will double the total forest biomass, M_{tot}, so that $M_{tot} \sim A$. Supplementary Equation 11-13, assume a constant area, A. As area, A, is a simple multiplier on M_{tot} we can also incorporate the additional multiplicative effects of *both* area and maximum plant size so that we have

$$M_{tot}^{plant} \propto \left(m_{max}^{\frac{5}{8}}\right) \cdot A \qquad (15)$$

and given the above relationships for animals we would have

$$M_{tot}^{animal} \propto \left(m_{max}^{\frac{5}{4}}\right) \cdot A \qquad (16)$$

Supplementary Eqn 15 predicts that efforts to increase area and maximum plant size will have a nonlinear effect on forest biomass and carbon stocks. Similarly, efforts to increase the maximum animal size protected will increase total trophic biomass, Supplementary Eqn 16. So, together, allowing for increases in maximum organismal size (conserving and promoting growth and survival of the largest tree sizes and allowing or large animals allowed to survive) and allowing an increased area to be restored to forest or habitat will have a multiplicative nonlinear effect on the total amount of carbon and biomass stored in forests and animal trophic levels. Interestingly, plants and animals differ in terms of their scaling functions. Larger animals have more of a disproportionate impact. While the total amount of animal and plant biomass will both increase with the size of the largest individual, biomass in animal communities are predicted to increase faster with maximum size so will contain disproportionately more biomass (because of the 5/4 exponent) as the size of the largest individual increases. In other words, within a given area, loss of the largest plants and animals will reduce the total trophic biomass. However, the loss of large animals is predicted to have a more disproportionate impact on heterotrophic biomass than autotrophic biomass. These predictions are shown graphically in Supplementary Figure 1.

We know of no study that has assessed the multiplicative impact of conserving both the size of the largest organism and the role of conserved area.

Summary of Predictions. The above equations make several predictions for how the size downgrading of the biosphere with the loss of the megabiota will influence ecosystem functioning. Specifically, communities, landscapes, and ecosystem with larger plants and animals will have larger stocks and flows. In general, metabolic scaling theory and empirical data show that communities and ecosystems with larger body sized plants and animals store more carbon and nutrients, flux more energy and resources, and are more fertile. Thus, continued reductions in body size will lead to continued reduction in ecosystem stocks and flows. Reductions in the largest organisms is predicted to reduce the amount of carbon and nutrients stored in ecosystems and lead to a reduction in productivity, biosphere metabolism, and lead to a reduction in the fertility of the biosphere.

The Madingley General Ecosystem Model (GEM).

The conceptual basis for General Ecosystem Models (GEMs) is described by Purves et al.[98] and a comprehensive explanation of the GEM are provided by Harfoot et al.[99] . Here, we summarize key terrestrial components important to our analysis and outline fundamental improvements made to the infrastructure of animal feeding ecology.

The Madingley model GEM explicitly simulates the dynamics of plants and all heterotroph organisms between 10 μg and 150,000 kg. The model is mechanistic, generating emergent ecosystem structure and function by simulating a core set of biological and ecological processes at the level of an individual. The GEM is global in scope, but is spatially and temporally flexible, allowing for application at regional and local scales and can be applied in both the terrestrial and marine realms.

On land, plants are represented by stocks of biomass simulated using the climate-driven model of Smith et al.[100]. Plant biomass is added to the autotroph stock each time step in each grid cell through environmentally-driven primary production, the seasonality of which is calculated using remotely-sensed Net Primary Productivity or NPP. This production is allocated to above-ground/below-ground, structural-non-structural and evergreen/deciduous components as a function of the environment. Biomass is lost from plant stocks through mortality from fire, senescence and herbivory.

For computational reasons, individual multicellular animals cannot be represented uniquely in the Madingley General Ecosystem Model (GEM) - Purves et al.[98] estimate that it would take 47 billion years for a standard laptop to model every multicellular animal within a 1-degree grid cell for 100 years. Instead, heterotrophic animals are grouped into cohorts, or collections of individuals within a grid cell with identical traits. These traits are both categorical, including feeding mode (herbivorous, carnivorous or omnivorous), metabolic pathway (endothermic or ectothermic) and reproductive strategy (iteroparous or semelparous) and continuous, including juvenile, adult and current body mass. Heterotroph dynamics result from six key processes applied at the individual-level; metabolism, predation, eating, reproduction, dispersal and mortality as represented in Supplementary Figure 1. Using this approach, the Madingley GEM can simulate complex networks of ecological interactions across scales from individuals to entire ecosystems. The emergent properties of individual organisms and the coarse structure of ecosystems resulting from these interactions have been demonstrated as broadly realistic in both the terrestrial and marine realms [99,101].

The importance of organismal body size in influencing metabolic demands, foraging area, and population dynamics provides a mathematical foundation to explicitly integrate the effects of allometric and metabolic scaling relationships on complex, emergent trophic interaction networks (for further details see Harfoot et al.[99]).

Mass-based feeding order.
As described by Harfoot et al.,[99] cohorts act sequentially within a given time step and the order in which they act is drawn randomly. Although this randomization does remove apparent bias, our subsequent research has revealed that there are size dependent biases from the structure of the model that emerge in experiments where simulated ecosystems are perturbed or altered and compared to initial conditions. In the case of herbivory, all cohorts within a model grid cell compete for the same plant resources within a time step. Environmentally determined time step production is added to the plant matter available to herbivores at the beginning of a time step. Therefore, a cohort acting early in the ordering can encounter a high density of plant matter, whilst a cohort acting later in the ordering, after other herbivores have eaten, can encounter much lower densities. The feeding rates of model organisms are dependent on body size and the density of plant matter with larger organisms and greater plant matter densities leading to higher rates of consumption. Small organisms in low plant matter densities can have negligible feeding rates, whilst larger organisms may still consume some resources. As a result, in the random ordering of cohorts, when small organisms act later in the order they are more likely than larger organisms to eat insufficient food to balance their metabolic costs (which for smaller body sized organisms are higher per unit body mass compared with larger organisms) and therefore to suffer starvation mortality.

To avoid this selection bias, we amended the ordering scheme to one in which the order of action was weighted towards smaller organisms acting first. This weighted ordering is established by drawing a cohort, i, at random from the list of cohorts within a grid cell, and determining whether it acts, here X_i, as follows:

$$X_i = Bernouilli\left(1 - \frac{\log(M_i)}{\log(M_{max})}\right) \quad (17)$$

where M_i is the individual body mass of cohort i and M_{max} is the maximum mass of any cohort in the current grid cell.

Experimental constraints.

The complex network of dynamic ecological interactions modelled within the Madingley GEM model can lead to unpredictable behavior. Consequently, to constrain our analysis to a single cause we circumscribed our experiments to manipulating the body size of endothermic herbivores only. For this, we exclusively modelled the terrestrial realm because the megafauna extinctions have been less severe in the oceans than on land [102].

The constraining characteristics of cohorts used in the three experiments are summarized in Supplementary Table 1. For all cohorts except endothermic herbivores, these properties were kept as those used by Harfoot et al.[99], which are broadly realistic of the late Pleistocene prior to the megafauna extinctions [103]. We use the same annual time-series of monthly climatological input data for each year of the 100-year simulation, and importantly do not include the effects of anthropogenic habitat conversion or the harvesting of plants and animals. As a result, except for climatic conditions which are based on averaged data between 1961-2000, our simulations represent a late-Pleistocene ecological world.

We experimentally changed the maximum attainable body size of endothermic herbivores across two orders of magnitude from 10,000 kg to 100kg. This upper value is representative of the largest known herbivorous mammal, *Mammuthus columbi,* as estimated from the fossil record by Faurby and Svenning[103]. Importantly, these upper and lower body masses determine the possible range over which cohorts can theoretically be realised in each grid cell. Once the model is running, however, environmental and ecological pressures may not allow these limits to be reached.

GEM - Ecosystem Metric Derivation

Heterotroph Biomass.

The GEM is not deterministic, so we performed an ensemble of five 100-year global simulations for each world using a monthly time step and a resolution of 2° x 2° grid cells. After 100 model years simulations reached a dynamic steady state. Heterotrophic organisms were assigned to one of 25 mass bins based upon the natural logarithm of their individual body mass. To capture the seasonal variations in grid cell heterotroph biomass, we calculated the mean heterotrophic biomass in each grid cell using the last 12 monthly time steps, or one whole year. This was performed for the 5 simulations run for each world, from which an ensemble mean value was derived for each terrestrial grid cell. Heterotroph biomass within each grid cell and globally was then summarized by trophic group, thermoregulatory strategy and mass bin for use in our experimental analysis.

Heterotroph Metabolism.

The metabolic rate, in kJ/day, for each organism was calculated assuming a power-law relationship with body mass and an exponential relationship with temperature following [104]. Whilst an organism is active, the metabolism of a given individual, i, is described by field metabolic rates, M_i^{metab}, whilst when inactive metabolism is described by basal metabolic rates (see Ref[99]):

$$M_i^{metab} = \left[\begin{array}{c} \left(\varsigma_{f,(t)} . I_{0,f}^{FMR} . exp^{-\left(E_A / k_B . T^{K,body} \right)} . \left(M_{i(t)} \right)^{b_f^{metab,FMR}} \right) + \\ \left(\left(1 - \varsigma_{f,(t)} \right) . I_0^{BMR} . exp^{-\left(E_A / k_B . T^{K,body} \right)} . \left(M_{i(t)} \right)^{b^{metab,BMR}} \right) \end{array} \right] \quad (18)$$

where: $\varsigma_{f,(t)}$ is the proportion of the current timestep for which environmental conditions are suitable for cohort i, belonging to functional group f, to be active. Endothermic functional groups were considered to be metabolically active for the entirety of a time step with a body temperature of 310K. For ectothermic functional groups, the proportion of time active and body temperature was derived from the ambient temperature T^K.

Supplementary Figure 3 compares the modelled daily individual metabolic rate of organisms in the last (1199th) time step of a single Pleistocene world simulation to empirically derived data of field metabolic rate by Nagy et al. (1999)[105]. Overall, the metabolism of Madingley Ectotherms and Endotherms overlap with observed 'real' organismal metabolic rates listed in Nagy et al. The GEM endotherms show a consistent pattern with the 79 mammal and 95 bird species used in the Nagy et al. study. However, based on their body mass, the modelled ectotherms in our GEM have a consistently lower daily metabolic rate than the field metabolic rates as shown by 55 reptile species. This likely results from two reasons. The first is the use of mean ambient air temperature for the calculation of ectothermic metabolic rate. In practice, it is known that ectothermic species can behaviorally regulate their temperature above or below that of the ambient temperature[106] , a process that is currently not included in the Madingley model. The second, related issue is that in the model, depending on the environment, ectotherms might be inactive for a proportion of the day during which they metabolism at a basal metabolic rate. So, the total daily metabolism should always lie between basal and field rates. In addition, the GEM also does not include several important processes that pertain to endothermic metabolism, including fat storage[107], hibernation[108]) and responses to ambient temperature[109]. The metabolic rate calculated here is exclusively related to body mass, and as a result there is a perfectly linear relationship in Supplementary Figure 3. As for heterotroph biomass, heterotrophic metabolism was summarized within each grid cell and globally by trophic group, thermoregulatory strategy and mass bin.

Nutrient Diffusivity.
Nutrient diffusivity was calculated from a modified version of equation 3 in Wolf et al.[110]:

$$\phi = (1-\varepsilon)B\left(\frac{N}{\alpha M_{Tot}^{Plant}}\right)\frac{\left(A\cdot t_{gut}\right)^2}{2\cdot t_{gut}} = 0.050\ m_{Herbivore}^{1.17} \tag{19}$$

Where N is population density (#/km^2), B is metabolic rate (watts), A is day range (km), and t_{gut} is the gut passage time (days). The term αM_{Tot}^{Plant} is an edible autotrophic biomass term (which is not modified in our simulations) and ε is the fraction incorporated into the body. Wolf et al (2013) found a nutrient diffusion capacity, $\phi = 0.053\ M^{1.011}$, which is estimated by substituting the allometric scaling of PD, MR, DD, and PT. In our paper, we start by using a slightly different version of the equation for ϕ in other parts of this paper ($0.050 * M^{1.17}$), which stems from a statistical fit to primary data. A 95% confidence interval of this coefficient was previously calculated as ± 0.24 (see Ref [81,111]). Next, because our GEM can calculate animal density, we then calculated $\Phi_{excreta}$ by inputting animal density predicted by our GEM into Supplementary Equation 19. This required us to remove our previously used N value calculated using primary data $N = 87.6(M^{-0.724})$ (see Ref[110]) from our previously calculated $\phi = 0.053\ M^{1.011}$. Therefore, our expanded equation 3 from Wolf et al (2013) now includes PD and mass, M as separate independent variables calculated from our GEM so that,

$$\phi = N * (\ 0.000605\ \ M^{1.735}\ ) \tag{20}$$

We assessed how sensitive our estimates of global nutrient diffusivity (ϕ) are to variation in the scaling of food passage time, PT. In this sensitivity analysis, we also calculated Supplementary Eqn

4 using our original allometric function calculated from primary data ($0.050*M^{1.17}$), and also a value calculated with a lower possible value for food passage time or t_{gut} where $t_{gut} \propto M^{0.14}$(see Ref. [112]) instead of the canonical $M^{1/4}$. Therefore, in addition to Supplementary Eqn 20, we used 6.05e-04*$M^{1.60}$ as a lower bound estimate of ϕ and 5.70e-04*$M^{1.89}$ as an upper bound estimate of ϕ.

We limited our calculations for this metric to endothermic cohorts only. This is because endothermic organisms are considered the major vectors of lateral biotic nutrient movement and the coefficients used in Supplementary Eqn 20 pertain to empirically derived mass-scaling relationships based on endotherms alone[66,110]. Consequently, the use of these mass-scaling relationships for ectothermic cohorts would have overestimated total nutrient diffusivity.

Supplementary Results

Regional heterotrophic biomass

Our modelled biomes harbor markedly different abundances and forms of heterotrophic life with concomitant implications to ecosystem structure and function. Greatest heterotrophic biomass is modelled in sub-tropical regions of the world with endothermic cohorts contributing vastly to the total heterotrophic biomass in these regions (Supplementary Figure 2 c,e). There is very low heterotrophic biomass in arid regions (e.g. Sahara and Gobi deserts), and life here is strongly weighted to larger endotherms cohorts (Supplementary Figure 2 f), which correlates with empirical evidence for both mammals and birds[113,114]. In the tropics, there is a large increase in ectothermic life, with biomass peaking around 0.1 - 5g (Supplementary Figure 2 b). This mirrors the high level of insect life found in tropical forests. There is evidence for bi-modal distributions of endothermic biomass in A, C and E, with a secondary peak in biomass occurring at around 1kg. However, this bi-modality is most pronounced in eastern Europe with peak biomass peaks between 1 - 20 kg and 300 - 1000 kg (Supplementary Figure 2 d). This suggests two broad classes of successful life history strategies in endotherms; be big and escape predation pressure and survive suboptimal seasonality periods, or be small and rapidly take advantage of optimal conditions for population expansion.

GEM maps of predicted heterotroph metabolism, nutrient diffusivity and autotroph biomass

Heterotroph metabolism. The pattern of heterotroph metabolism broadly follows that of heterotrophic biomass. However, regions where animal biomass is weighted towards smaller mass bins, such as the peak of endothermic biomass between 1 - 20kg in Europe, generates higher regional heterotrophic metabolism (Supplementary Figure 4 a). In the Modern and Future worlds, heterotrophic metabolism is reduced in regions where total biomass is lost, most notably in the sub-tropics. Tropical regions have spatially heterogeneous changes of heterotrophic metabolism with some cells showing small both increases and decreases in heterotrophic metabolism (Supplementary Figure 4 b,c).

Heterotroph nutrient diffusion. High heterotrophic nutrient diffusivity mirrors the geography of large herbivores. Sub-tropical regions in SE South America, SE Asia and E Austria harbor the highest levels of nutrient dispersal, whilst in arid and high-latitude regions, nutrient diffusion is very low (Supplementary Figure 4 d). The reduction of maximum attainable endothermic herbivore body size in the Modern and Future worlds significantly reduces these high nutrient diffusion regions (Supplementary Figure 4 e,f).

Autotroph biomass. The broad patterns of autotroph biomass - which is referred to here as leaf biomass available to consumption by herbivores and omnivores - conform to those observed via satellite imagery (Running et al., 2004; Supplementary Figure 4 g). In our experimental worlds, the removal of megaherbivores leads up to a ~50% increase in autotrophic biomass in the regions of high megaherbivore density in the Pleistocene world. There is little change to the annual mean stock of autotroph biomass in grid cells outside of these regions (Supplementary Figure 4 h,i).

Global ecosystem measures summarized by trophic group (Supplementary Figure 5). Reducing the maximum attainable body size of endothermic herbivores most significantly affects the total biomass in that trophic group. In our Future world simulations, the total biomass of endothermic herbivores globally is 21.8% when compared to the Pleistocene simulations (Supplementary Figure 5a). The direction of change across the other trophic groups is small, and non-uniform. The

contribution of each trophic group to global heterotrophic metabolism varies substantially between tropic group. Endothermic herbivores and omnivores dominate, whilst ectothermic carnivores at the global scale contribute negligibly to the total metabolism, despite harbouring a similar magnitude of heterotrophic biomass to the other trophic groups excluding endothermic herbivores. Nutrient diffusivity is overwhelming dominated by endothermic herbivores and following the removal of these cohorts in our experimental Future world, this ecosystem metric for endothermic herbivores is reduced to 1.3% of the Pleistocene value.

Nutrient sensitivity tests (Supplementary Figure 6). Our sensitivity tests for nutrient diffusivity show a high degree of divergence from that calculated in Supplementary Equation 4 for both the lower and upper bounds, underling the importance of scaling coefficients to this metric (Supplementary Figure 6). However, despite the magnitude of change to nutrient diffusivity in our sensitivity tests being large, we obtain similar conclusions to the full model. When we used the lower bound for the scaling of gut passage time, $PT \propto M^{0.14}$ (see Ref. [112]), the shallower scaling of PT leads to a reduction in global diffusivity estimates for each of the simulated worlds. This was found to be equal to 38%, 45% and 50% of our values calculated using Supplementary Equation 4 in the full model for the Pleistocene, Modern and Future worlds respectively. In contrast, when we calculated an upper bound, using the steeper exponent for nutrient diffusivity based on Wolf et al.[110] , we found that global diffusivity was 295%, 238% and 216% of our values in the full model for the Pleistocene, Modern and Future worlds respectively (Supplementary Table 2). Nonetheless, our results indicate in both upper and lower estimates of the scaling of PT there is a large decrease in global nutrient diffusivity with reductions in the megabiota.

Spatial variation in abundances and impact of reduction of heterotrophic biomass (Supplementary Figures 7 and 8). Our simulations show wide spatial variation in heterotrophic animal abundance and the percent reduction in heterotrophic biomass. These analyses emphasize the spatial variation of impact of percent reduction (more of an impact in areas that harbored a larger body size range to start with). These percent change maps for the modern and future worlds show that the percent reduction is greatest in savanna/grassland/desert environments and less in forested (tropical and boreal) regions. As a result, geographic variation in the global impacts of the reduction in body size ranges modulate the predictions given by Metabolic Scaling Theory or MST. Because of climate, different parts of the planet vary in terms of the number and original size range of heterotrophs (see also Supplementary Figs. 4 and 5). Some parts of the planet originally harbored more larger herbivores than other parts of the planet. As shown in Figure 5C reductions in biomass and productivity are not uniform across the globe. As a result, in our simulations, there are large areas of the globe that do not experience the dramatic reduction in body size ranges. The reduction of biomass observed in Table 1 are total heterotrophic values across all animal groups across all biomes across the world (i.e. global averaging to capture feedbacks and interactions). Focusing on areas within the endothermic herbivore trophic group or in places with high levels of megaherbivores we see larger effects when we reduce the size of the largest animals for both biomass and metabolism.

Supplementary Discussion

The potential role of ecological compensation of the smallest plants and animals. We used the Madingley model to assess if compensatory dynamics of smaller organisms can compensate or buffer the loss of the megabiota. Such potential compensatory effects reflect complicated ecological interactions and responses. For example, smaller organisms could respond by increases in population density, shifts in competitive interactions and/or resource use. Reductions in the megabiota could also impact resource supply and shift the shape and functional form of the size distribution (for example the size distribution in Figure 2 could shift more than is diagramed). While we found some evidence of compensation (see Figure 5E, where we see increases in the metabolism of smaller sizes classes) the degree of compensation, however, was not of the magnitude for complete compensation for ecosystem and biosphere metabolism, productivity, and fertility (see our Figure 5).

The next steps in assessing the role of compensatory processes. A critical next step in the development of theory to scale from individuals to ecosystems will be to better include ecological compensation to the loss of larger plants and animals. The original metabolic scaling theory or MST papers (deriving the nature of the size distribution and spatial packing of individuals) detail how size influences the number of individuals per unit area. Each size class uniformly fills space, independent of the other size classes. While smaller individuals are packed closer in space, larger individuals are further apart (see Figure 2 and Supplementary Figure 1[68]). In the theory, competition for space is with similar body sizes (not larger or even smaller body sizes). So, under the canopies or home ranges of the largest individuals, individuals of smaller body sizes exist underneath or within (again see Fig. 2 in West et al. 2009). As we discuss below, however, removing larger size classes can potentially free up additional resources available to the smaller individuals and potentially increase population density (see Equation 10 in Enquist et al. 2009). In principle, the size distribution can change in response to loss of a given size class via the influence on resource supply rates to surviving size classes. For example an increase in resources to smaller size classes can increase the number of individuals in that size class and potentially change the community size distribution exponent.

An experimental field test of forest compensatory responses to the loss of the megabiota. Recently, Riutta et al. [69] investigated how selective harvesting of the largest trees impacted forest size structure, total biomass, and net primary productivity. In support of the space filling assumptions of MST (see above) they did not find a notable shift (see their Fig. 2) in the shape of the size distribution despite the loss of the largest trees. The number of smaller trees in forests of selectively logged forests (where the largest trees were removed) did not increase in abundance. In short, analyses by Riutta et al. (2018) does not show a notable shift or compensation in the number of small individuals in the size distribution. Nonetheless, like our GEM simulation, Riutta et al. (2018) do show that some compensatory dynamics in the smallest size classes did occur.

The Riutta et al.[69] study did not find total compensation in total forest biomass but did find evidence of compensation in net primary productivity or NPP. The total biomass of forests that lost their largest individuals was significantly lower than old growth forest with its large trees intact. This is consistent with our theory that predicts that loss of largest individuals will decrease total forest biomass (especially if there is no compensation in the smallest individuals). However, this study does evidence in compensation in whole forest NPP. Forest disturbance and removal of large individuals can yield a compensation dynamic that can influence NPP via possibly influencing the age structure. Indeed, the removal of the largest trees in a tropical forest can cause a compensatory

effect by causing shifts in carbon production and allocation in tropical forests (Riutta et al. 2018) so that NPP in selectively logged forest was similar to old growth.

The results of Riutta et al.[69] are consistent with previous work extending metabolic scaling theory where disturbance (or selective logging) can influence the 'age' of a stand. Older forests, for their biomass, do have a lower productivity (see Ref[58,59]. However, this age effect is smaller than the tree size effect (see[58,59]; see also Fig. 3B). Removing large trees will lead to a reduced total forest carbon and biomass but that compensatory dynamics via a forest age effect (as shown in Fig. 3) can modify/compensate for forest productivity (Fig. 3B) but that we expect that this 'age' effect is on the order of ⅓ of the effect of loss of the largest trees. Riutta et al. (2018) indicate that this 'age effect' is due to directional shifts in the growth efficiency and possible supply of soil nutrients. Thus, the megabiota effect in forests is expected to primarily impact total biomass (stored carbon) and to a somewhat lesser effect on net primary productivity mainly due to the age effect as younger regenerating forests can partially compensate for loss of larger trees. Nonetheless, as noted in [58,59] this compensatory 'age' effect across terrestrial vegetation is expected to be about a third of the effect of maximum plant size alone.

Implications for ecosystem trophic stability and species diversity.

A long and growing literature support our central conclusions of the importance of the megabiota by pointing to the importance of the megabiota to maintaining and promoting biological diversity and ecosystem functioning[70]. The loss of megabiota result in major effects on vegetation and ecosystem functioning[70] via changes in species interactions[71], seed dispersal[72,73] and nutrient biogeochemistry [66,74]. Until recently it has been difficult to attribute ecosystem changes to reductions in the megabiota because little is known about their behavior. Recent studies demonstrate that in landscapes that still harbor large animals the larger animals play a key role in maintaining tree diversity and ecosystem function[75–78]. For instance, for many large-seeded fruit types, passing through the gut of elephants and other mega-fauna can improve germination and reduce seed predation[76,77]. Increasing numbers of studies have highlighted that the loss of large predators leaves a characteristic signal of recruitment failure [79] in the dominant tree species and reduced tree growth rate[80]. Further, loss of seed dispersing larger animals is predicted to result in a slow range collapse and ultimately extinction of plant species that have evolved adaptations for dispersal by large animals. Analysis of the geographic ranges of numerous megafauna dispersed trees in South America reveal significantly smaller geographic ranges [81] supporting the prediction that loss of large animals will lead to the reductions of geographic ranges of plants that are dispersed by large animals.

Recently, Estes et al.[71] have underscored that ecological theory based on species trophic interactions has predicted that major shifts in ecosystems can follow changes in the abundance and distribution of large apex consumers[82,83]. This trophic downgrading of the planet can result in several cascading effects. Reductions in animal population densities and animal total biomass can then ramify to influence the total plant (autotrophic) biomass by either or increasing or decreasing vegetation biomass[84]. Large animals can have major impacts on the vegetation structure, including the balance between trees and grasses, and increasing water and nutrients available for the growth of large trees in forests[70].

One of the primary effects of removing large animals from ecosystems on species diversity is that the dispersal ability of fruits, seeds, across landscapes is reduced[85,86]. Decreases in dispersal expose more seeds and seedlings to environments of high conspecific density and thus high mortality [86]. The net result, is that tree abundance declines due to decreases in seed dispersal are slowed or even arrested. Theoretical insights using basic principles of population and community ecology point that continued reduction of large animal seed dispersers can result in abrupt vertebrate-mediated

feedbacks if fruit or seed availability drops beneath critical thresholds for consumer population persistence leading to reductions in plant species abundances and geographic distributions[85,86]. The reduction of seed and element dispersal has led to reduced biodiversity[85,86].

Reductions in large animal population densities, top carnivores, often results in dynamical ramifications known as "trophic cascades". During trophic cascades, the propagation of impacts by consumers on their prey ramify downward through food webs[87]. In short, for any ecosystem, large bodied animals exert strong cascading effects on ecological interactions. Indeed, Estes et al. assert that most ecological surprises that have confronted humanity including pandemics, population collapses, and species eruptions of pests, major shifts in ecosystem states, the rise of exotics and species invasions were spurred by changes in role or presence of large organisms. A review of recent ecological studies assessing the loss of large animals supports long-standing ecological theory about the role of top-down forcing in ecosystems but also highlights the unanticipated impacts of trophic cascades on processes as diverse as the dynamics of disease, wildfire, carbon sequestration, invasive species, and biogeochemical cycles[71].

Implications for disease and human health.

Several recent studies have highlighted that host/pathogen interactions and the emergence of new infectious diseases can be linked to the reduction of the megabiota. First, reduction of larger top predator animals have been linked to a change in the prevalence of disease[88] via increasing population density of the smallest animals. For example, predatory vertebrates indirectly protect human health by reducing population size of many rodent spread diseases[88]. Indeed, our Madingley model simulations seem to support these observations. Our simulation results (Figure 5) indicate that small mammals (rodent sized) increase in dominance (biomass) and appear to show a compensatory increase in energy flux through the biosphere. Megaherbivores can also indirectly impact disease through their impact on vegetation structure. One study found that the loss of African megafauna increased rodent-borne disease partially because changed landscape structure following the removal of ecosystem engineers as species like elephants create better habitat for rodent and pathogen populations[89].

Second, extensions of Metabolic Scaling Theory to host pathogen interactions suggests that a loss in the megabiota will also influence pathogen dynamics[90,91]. Body size of the host appears to be a critical trait in influencing pathogenesis. Perhaps the most important but yet little studied aspect of pathogen dynamics is that it and the cellular immune response of the host depend on the body size of the host [91,92]. Because the average metabolic rate of cells is typically lower in larger species the dynamics of pathogens and pathogenesis on the landscape are hypothesized to be influenced by body size of the largest organism. This is expected for three reasons,

(I) The host metabolic rate will affect the rate at which cells synthesize DNA and proteins which in turn could then influence the replication rate of viruses and the speed at which pathogens multiply.

(II) The cellular immune response of the host could be affected by allometric scaling as many physiological aspects of immune response (cell replication rates, T cell memory, will scale with metabolic rate of the host).

(III) Interactions between the replication rates of pathogens and the dynamics of immune responses could result in differences in the time to disease progression between host species. For several pathogens disease progression to symptoms and to death in a small set of pathogens in different host species scales with $\sim M^{1/4}$. As a result, dynamics for several diseases that affect humans (anthrax, Pseudorabies Virus, Transmissible

Spongiform Encephalopathy, Rabies, West Nile Virus and possibly HIV and SIV) are influenced by the body sizes of hosts present [91,93,94].

These results point that host body size and metabolic rate can constrain rates of pathogenesis and pathogen evolution[90]. The number of parasite and potentially species colonizing mammals is likely to scale positively with body size and mass scaling relationships suggest that the largest extinct megafauna species could have hosted a wide diversity of parasites species. With the loss of the megabiota human health can be impacted because disease dynamics and pathogenesis is expected to change due to reductions in the range of body sizes.

We hypothesize that the reduction of body size will be associated with a shorter time until disease emergence [91] and reductions in the stability of pathogen/host interactions[90]. The reduction of dispersal of microbes and viruses across landscapes following the loss of the megabiota may also increase the likelihood of emergent diseases. Reductions in in dispersal abilities of pathogens associated with the loss of large hosts is likely also transforming human health by reducing gene flow between pathogen populations and separating ecological interactions that have evolved in the presence of the megabiota. For example, animals that are large body sized hosts may provide more of a role of limiting the rate of disease spread and evolution[94]. In contrast, diseases restricted to infect only smaller species may be highly competent at spreading the disease. Smaller species are likely more of a pathogen reservoirs and larger species are potentially more dead-end hosts [94]. These body size influenced interactions have tended to stabilize host/pathogen interactions and the emergence of new diseases [92,95,96]. Indeed, a recent model predicted that with a global reduction of the dispersal of microbes and blood pathogens can increase the presence of emergent diseases. They showed that an inclusion of changes in dispersal ability could improve the prediction of emergent diseases [94].

Recently, a model used allometric scaling to predict decreases in mean distance travelled by fecal pathogens and endoparasites and found a globally averaged decrease in dispersal distance of a third and 15% respectively. This approach found that decreased dispersal distances associated with the loss of megafauna were correlated to the outbreak of emergent infectious diseases, suggesting that reduced dispersal distance over time allowed greater pathogen diversity in an island biogeography effect (Doughty et al in review).

Caveats

Deviations from MST assumptions. We expect that our predicted functions describing how reductions in the megabiota will impact ecosystem functioning will be also influenced by additional factors not explicitly modeled here. For example, the above analytical derivations for the scaling of ecosystem stocks and fluxes assume resource and demographic steady state[10]. Our model assumes that *all* mortality is a consequence of size-based density-dependent competitive interactions (thinning in the case of plants) and the rate of mortality depends on the rates of growth. This is basically the steady-state assumption. As mentioned above, however, there are other sources of mortality and many of these differentially affect individuals of larger size. Factors such as disturbance, harvesting, episodic recruitment etc. are not primarily influencing the size distribution and allometric scaling[11,97]. As discussed in Enquist et al. 2009, deviations from model-predicted baselines may allow quantification of density independent and size-selective mortality. Additional sources of mortality or limitations to recruitment could be included in the model as additional terms. In summary, variation in allometric scaling exponents and ecological and historical processes, however, will cause deviations from steady state assumptions will also cause deviations from these predictions.

GEM global simulation results. In each of our simulated worlds, heterotrophic organisms were assigned to one of 25 mass bins based upon the natural logarithm of their individual body mass. Our results show the global total of our three ecosystem-level measures for each of these mass bins. Following the removal of megaherbivores we do not see any significant increase in the total biomass of smaller organisms despite the resultant increase in available autotrophic biomass (see SI 2). This suggests that at the global scale, the abundance of our modelled animals <100kg is primarily constrained by carnivory and autotroph resource density rather than the total size of the global autotroph stock.

It is important to note that our choice of maximum body size for carnivores is influential in the outcome of these results through the scaling of optimal prey size with body mass. Accordingly, changes to this parameter will have concomitant ecosystem-wide ramifications that future research needs to explore further. Interestingly, we do see compensation in heterotrophic metabolism by the smaller body sizes. This observation likely reflects the fact that smaller organisms metabolize faster per unit biomass. Therefore, following the removal of the megaherbivores, some of the extra autotrophic NPP that is freed up is converted into energy rather than accumulated as animal biomass. The strong weighting of nutrient diffusivity to larger endothermic animals highlights the inability of smaller animals to make up for some of the unique properties of megaherbivores. To sum, our results here using the Madingley Model highlight the different environmental and ecological pressures on the large and small animals, which subsequently influences their biogeography, abundance and ecosystem functions.

Next step research questions in Megabiota research

1. How will climate change influence future body size distributions? Few studies have dealt with how global warming will influence changes in body size[115], especially for ectothermic animals and plants.

2. To what extent can smaller organisms compensate for the loss of ecosystem functioning linked to the loss of larger organisms[70,116]?

3. What are the ecosystem implications of declining ranges of body size[115]? Do terrestrial and aquatic ecosystems differ in susceptibility to downsizing[115]?

4. How will the dynamics of ecosystems and biodiversity change in a world with fewer megabiota[116,117]? Because large organisms are long lived and their population cycles are longer the presence of large bodied organisms can buffer ecological systems. Will ecological systems and human interactions with ecological systems (fisheries, forestry) become less buffered with time with loss of the megabiota?

5. To what extent do the "domesticated megabiota" (e.g. cattle; forest plantations) functionally compensate for the decline of wild megafauna and flora[116]? Under what circumstances (e.g. nomadic cattle pastoralism and wood harvesting versus industrialized farming and forestry) do they exert different influences on ecosystem processes?

6. Because host body size influences rates of pathogenesis[91], how have/will disease dynamics and pathogenesis change with reductions in the megabiota[93]? How will the proportional rise of the "domesticated megabiota" influence disease dynamics?

7. How do differences in the patchiness and total area protected interact with differences in body size ranges to influence ecosystem functioning, carbon storage, and nutrient cycling[111]?

8. How long will it take reforestation and restoration efforts to revive ecosystem processes promoted by large body sized species[118]? Analyses have indicated that it may take thousands of years to return to steady state following extinctions. Large animal extinctions have a very long-term impact but it is not clear if their impact be lessened or modified.

9. Can ‘rewilding’ efforts (the introduction of larger animals and plants back into degraded landscape [119]) effectively recover the historical influences of the megabiota on ecosystem functioning? How long will it take rewilding efforts to return to baseline levels?

Supplementary Tables

Supplementary Table 1: Total Characteristics of cohorts used in the three experimental simulations. The maximum attainable body mass for endothermic herbivores in each world is highlighted in bold.

Trophic Group	Thermoregul-artory Strategy	Reproduction Strategy	Min Body Mass (g)	Max Body Mass (g)		
				Pleistocene World	Modern World (1000kg)	Future World (100kg)
Herbivore	Endotherm	Iteroparity	1.5	**10,000,000**	**1,000,000**	**100,000**
Omnivore	Endotherm	Iteroparity	3	1,500,000	1,500,000	1,500,000
Carnivore	Endotherm	Iteroparity	3	700,000	700,000	700,000
Herbivore	Ectotherm	Semelparity	0.0004	1,000	1,000	1,000
Omnivore	Ectotherm	Semelparity	0.0004	2,000	2,000	2,000
Carnivore	Ectotherm	Semelparity	0.0008	2,000	2,000	2,000
Herbivore	Ectotherm	Iteroparity	1	300,000	300,000	300,000
Omnivore	Ectotherm	Iteroparity	1.5	55,000	55,000	55,000
Carnivore	Ectotherm	Iteroparity	1.5	2,000,000	2,000,000	2,000,000

Supplementary Table 2: Total global nutrient diffusivity using three different mass-based scaling coefficients.

Simulation Ensemble	Global Nutrient Diffusivity (10^7 km^2/day)		
	Lower Bound	Supplementary Equation 4	Higher Bound
Pleistocene World	1.144	3.007	8.865
Modern World (1000kg)	0.363	0.800	1.906
Future World (100kg)	0.113	0.226	0.488

Supplementary Figures

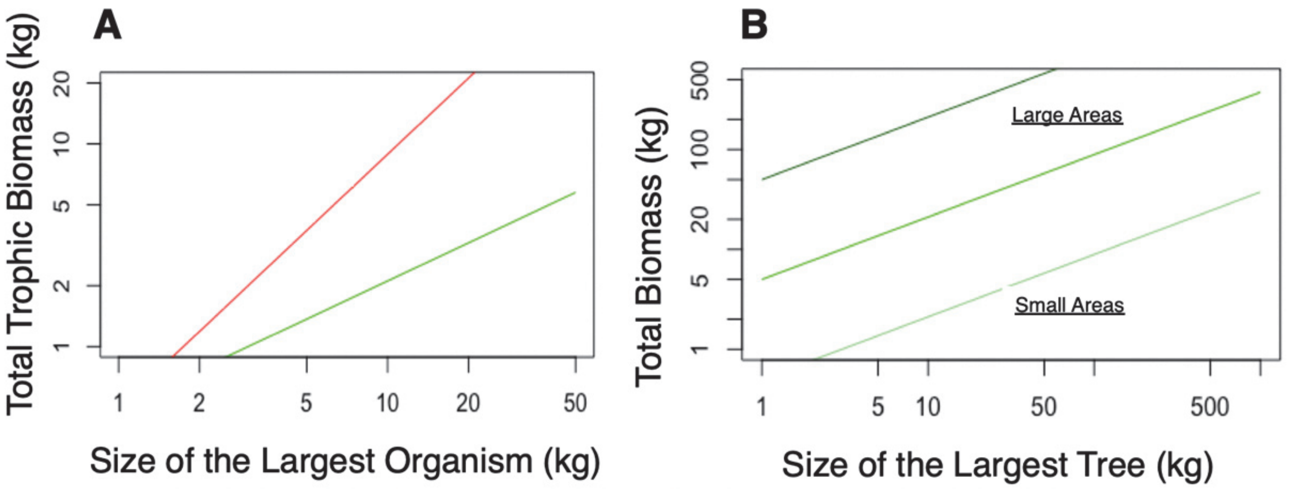

Supplementary Figure 1. Large organisms *and* large protected areas matter for conservation efforts prioritizing ecosystem functioning. Allowing for increases in maximum tree size or animal size *and* allowing more area to be restored to forest or to rewild large animals will together have a multiplicative and nonlinear effect on ecosystem services. This is viewed graphically three differing ways: **(A) Large organisms have a disproportionate impact -** Plotting how the total amount of autotrophic (green) and heterotrophic (red) biomass changes with the size of the largest organism. While the total amount of animal and plant biomass will increase with the size of the largest individual, animal communities (red) will contain disproportionately more biomass as the size of the largest individual increases. **(B) Large organisms *and* large protected areas matter** - Plotting how total autotrophic biomass changes with the size of the largest tree. Equations from larger areas hold more biomass (as expected) but forests with larger individuals will disproportionately hold more biomass the largest the size of the largest individual. Shown are forests that differ in their total area ranging from (5, 50, and 500km2). **(C) Landscapes with larger organisms will then tend to contain more biomass.** In other words, within a given area, loss of the largest plants and animals will reduce the total trophic biomass. However, the loss of large animals as in **A** is predicted to have a more disproportionate impact on heterotrophic biomass than autotrophic biomass.

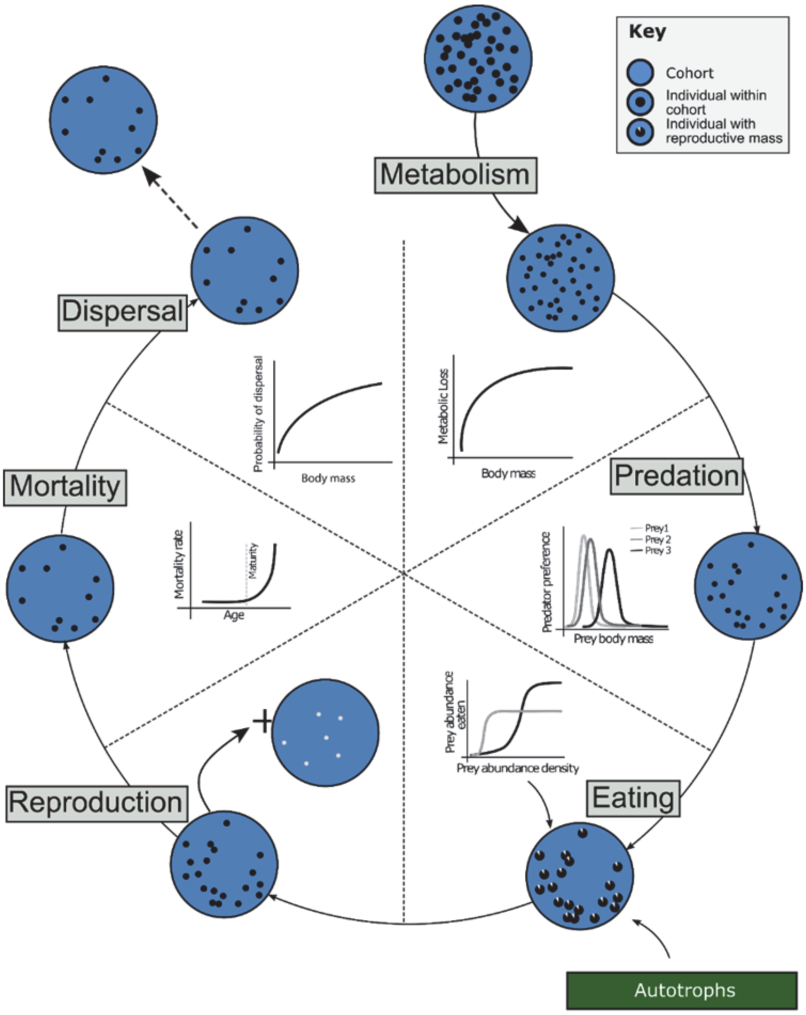

Supplementary Figure 2. Modelled ecosystem structure and function arises from six key biological and ecological processes operating on individual organisms within a grid cell.
Figure modified from Harfoot et al. (Ref[99]).

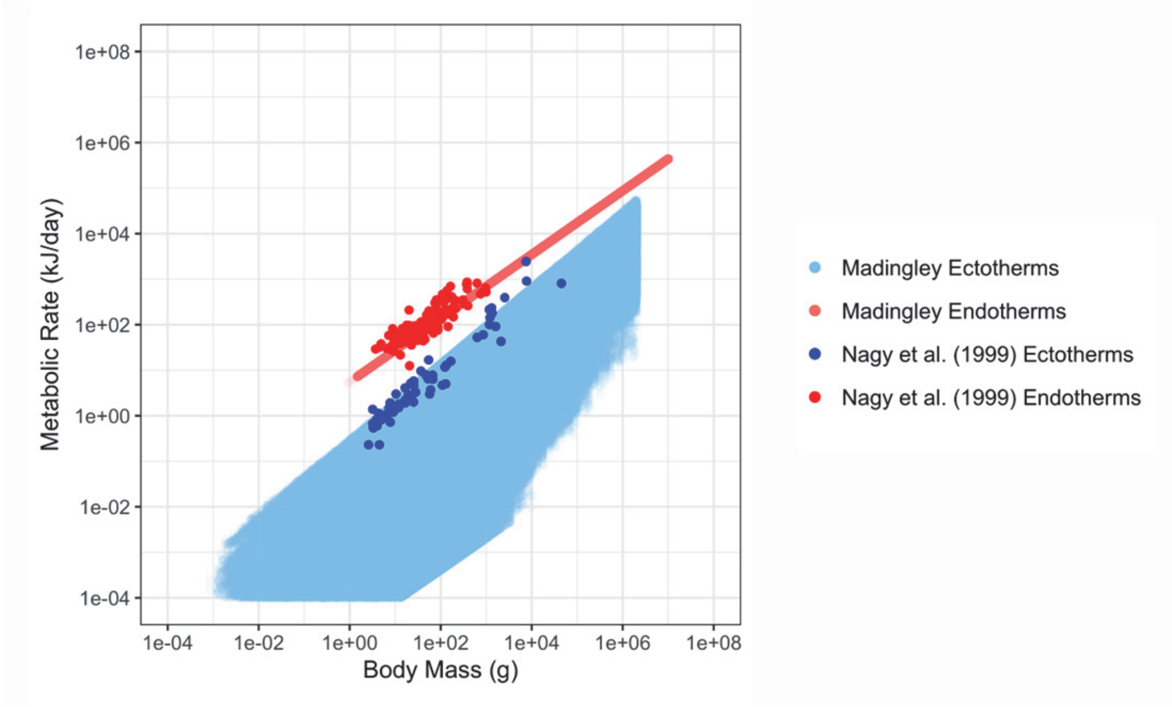

Supplementary Figure 3. Simulated individual organismal metabolism in the GEM compared with observed field metabolic rates from extant organisms. Simulated values calculated in the last (1199th) time step of a Pleistocene world simulation to empirically derived field metabolic rate from Nagy et al.[120].

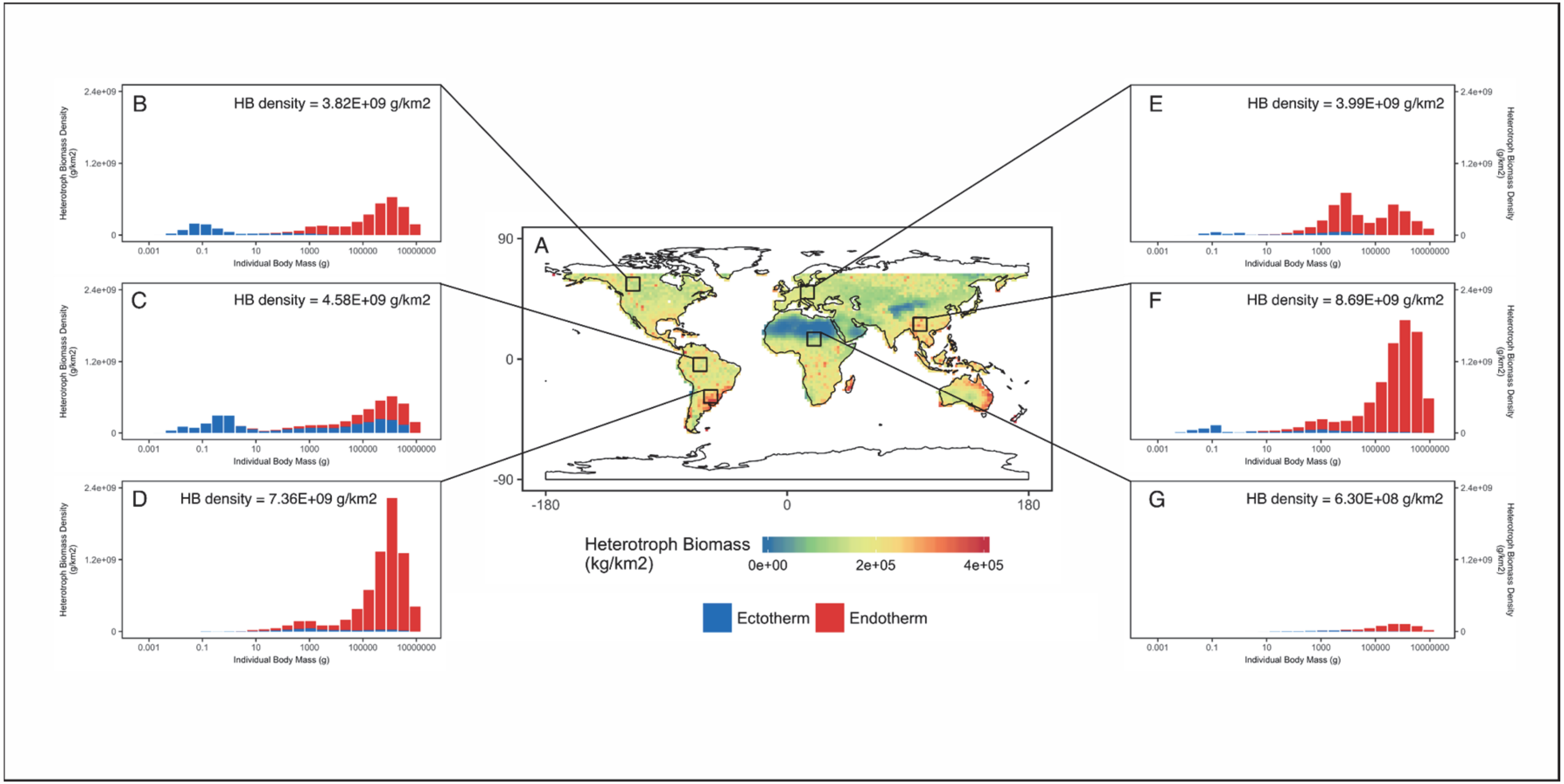

Supplementary Figure 4. (A) Mean heterotrophic biomass calculated over 12 monthly time steps, or one year, mapped spatially for the Pleistocene world ensemble. (B-G) Mean heterotrophic biomass for six 10o x 10o regions in the Pleistocene ensemble summarized by mass bin and thermoregulatory strategy. For comparison across latitudes, values have been converted to mean density (g/km^2) in each mass bin across each region. Across the globe, while there is variation in the magnitude of heterotrophic biomass, most heterotrophic biomass is in the largest size classes. This supports predictions from our theory which predicts increased total heterotrophic biomass in the largest size classes. Note: HB density refers to total heterotrophic biomass density across all cohort functional groups present in each region. Global map from the 110m land polygon shapefile from Natural Earth Data (https://www.naturalearthdata.com/downloads/110m-physical-vectors/).

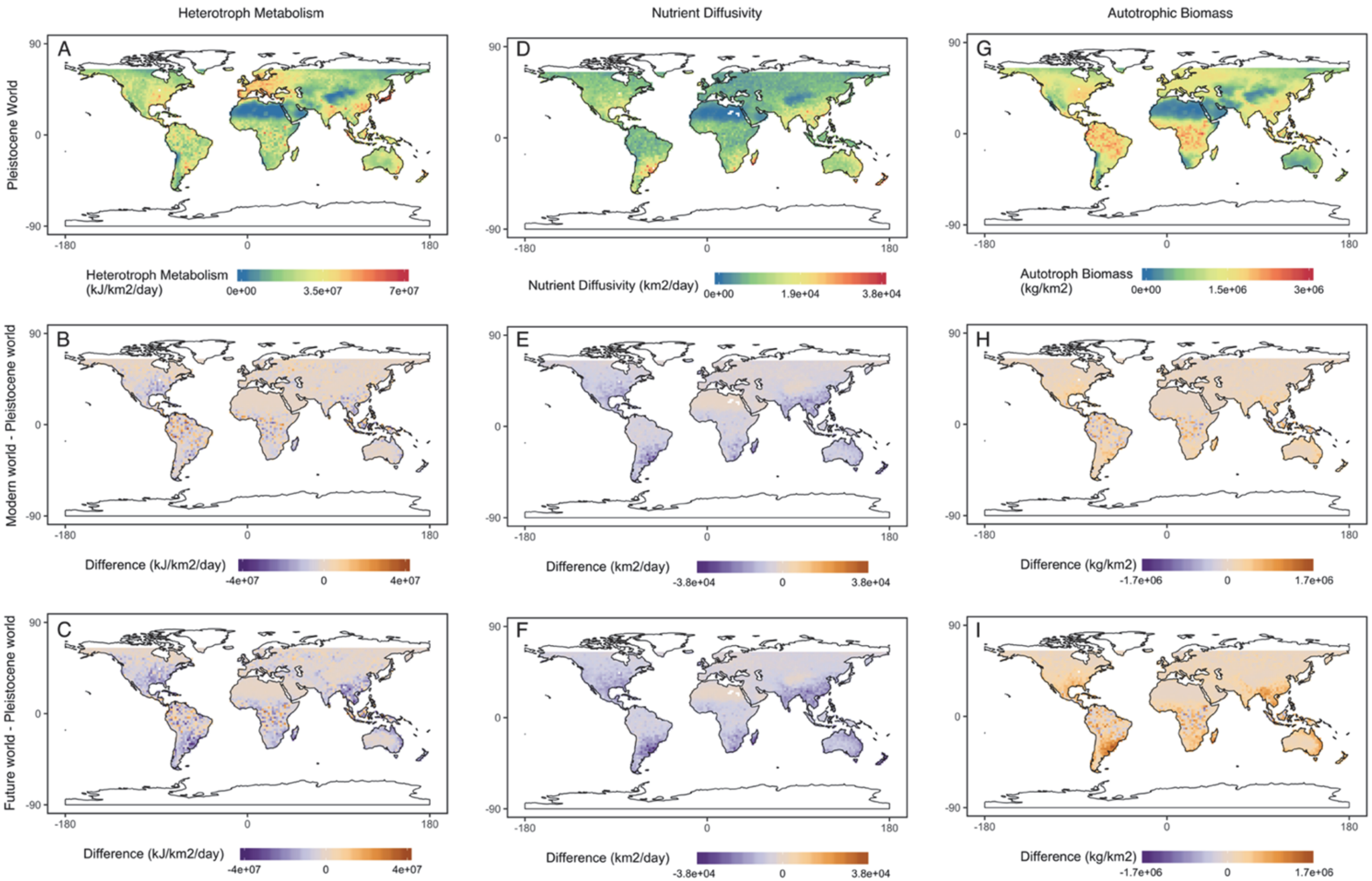

Supplementary Figure 5. Annual mean of three ecosystem metrics from the 3 ensemble experiments using the Madingley General Ecosystem Model (GEM) mapped spatially. ABC refer to heterotrophic metabolism, DEF to nutrient diffusivity and GHI to autotrophic biomass. Panels A,D and G exhibit these metrics for the Pleistocene world ensemble, whilst panels B,E and H represent the difference for each metric between the Pleistocene world ensemble and the Modern world ensemble, and C,F and I for the difference between the Pleistocene world ensemble and the Future world ensemble. Global map from the 110m land polygon shapefile from Natural Earth Data (https://www.naturalearthdata.com/downloads/110m-physical-vectors/)

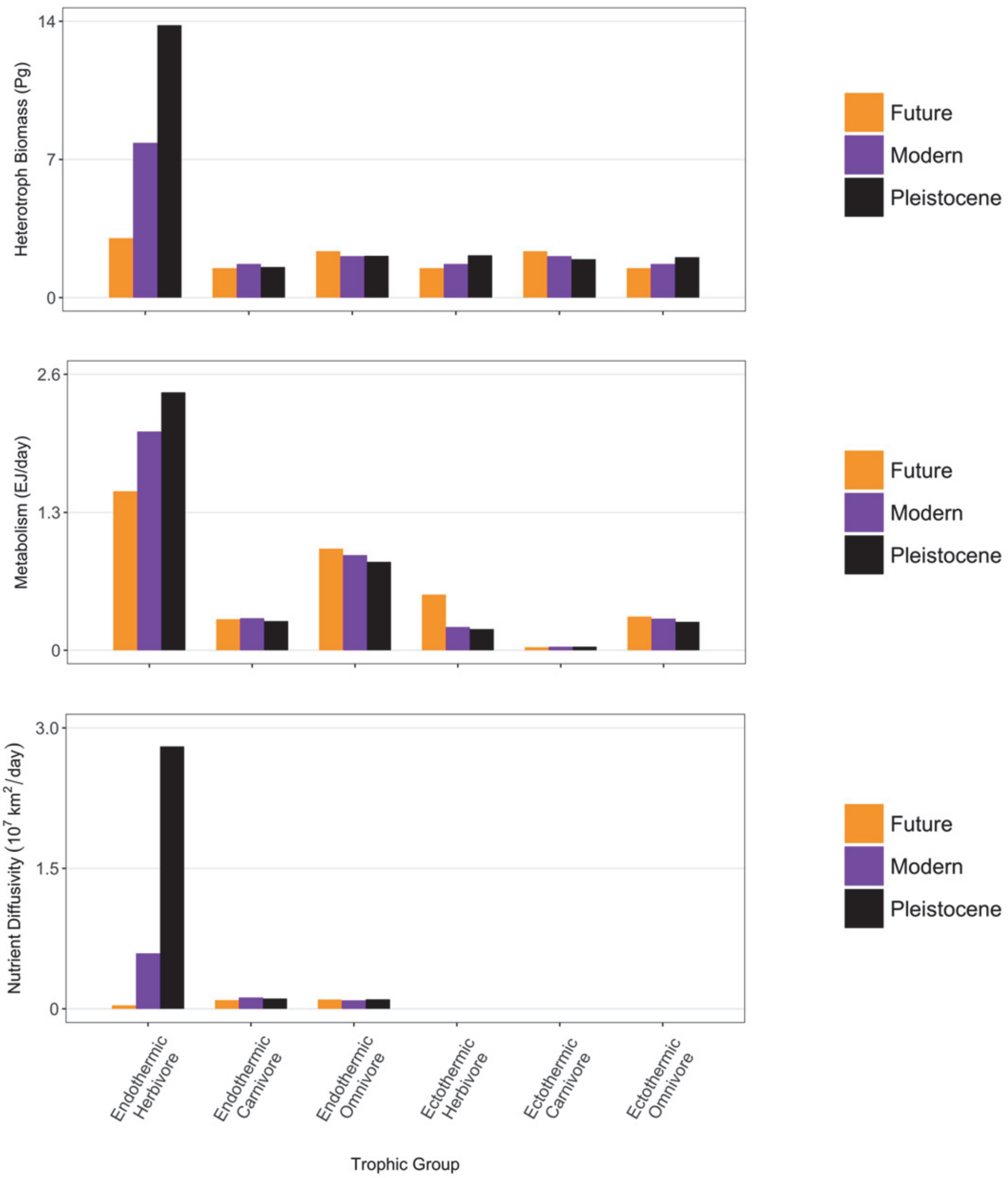

Supplementary Figure 6. Summarized into endothermic and ectothermic trophic groups. Annual mean (Top graph) heterotroph biomass (Middle graph), heterotroph metabolism and nutrient diffusivity (bottom graph) calculated across the three world ensembles.

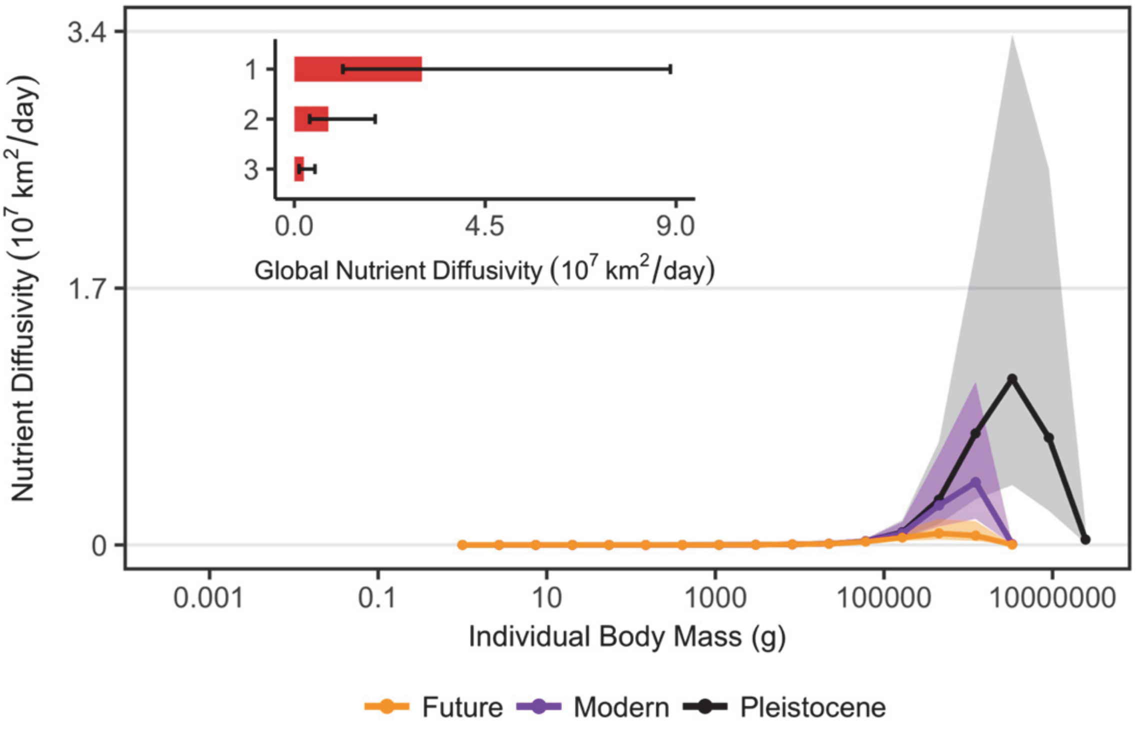

Supplementary Figure 7. Nutrient diffusivity sensitivity tests summarized into 25 mass bins for each world simulation. Error bars in the inset graph are 95% confidence intervals. Shaded areas represent the difference between the higher bound and lower bound tests for each mass bin. The inset graph displays the global total nutrient diffusivity with sensitivity error for each world, numbered 1) Pleistocene, 2) Modern and 3) Future world respectively.

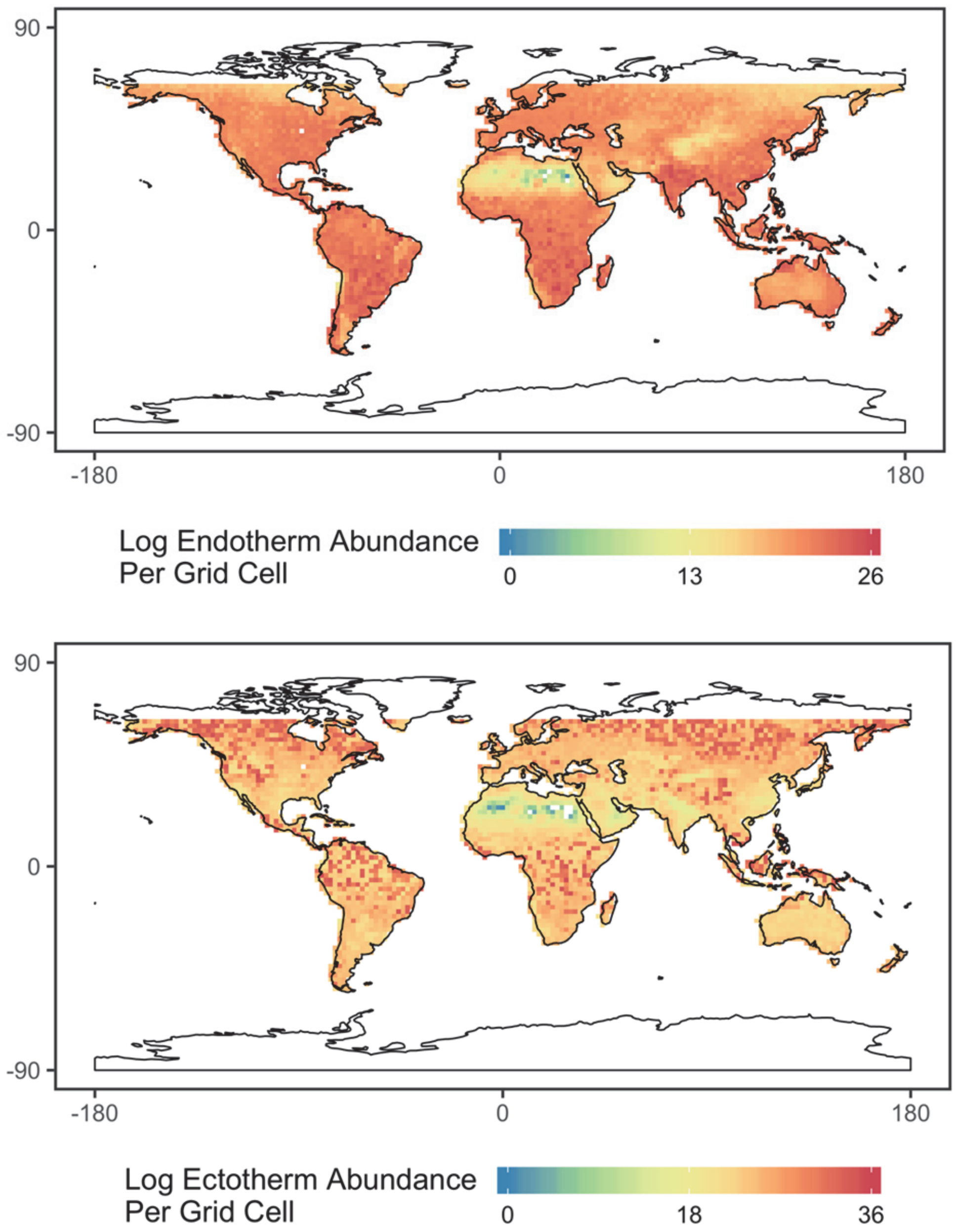

Supplementary Figure 8. Global variation in the total number of simulated heterotrophic individuals. The total number of individuals in each grid cell for endotherms (top) and ectotherms (bottom) for the last timestep from one of our Pleistocene world simulations. Note, abundances of ectotherms are generally higher in forest regions, especially in tropical and boreal forests. Endotherms, however, tend to be more uniformly distributed throughout the globe except for the most arid environments where abundances tend to be lower. Global map from the 110m land polygon shapefile from Natural Earth Data (https://www.naturalearthdata.com/downloads/110m-physical-vectors/)

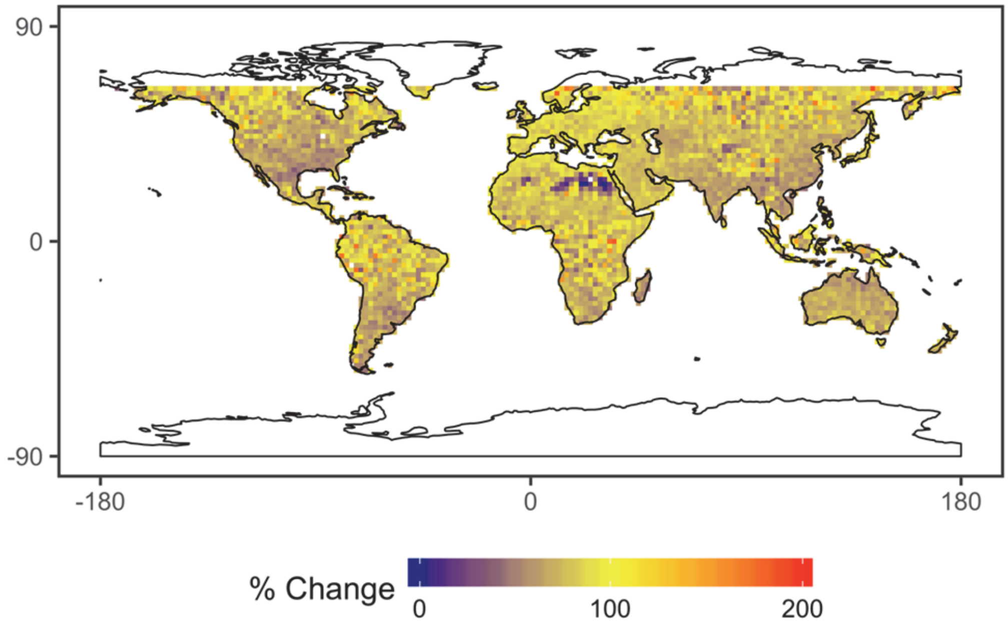

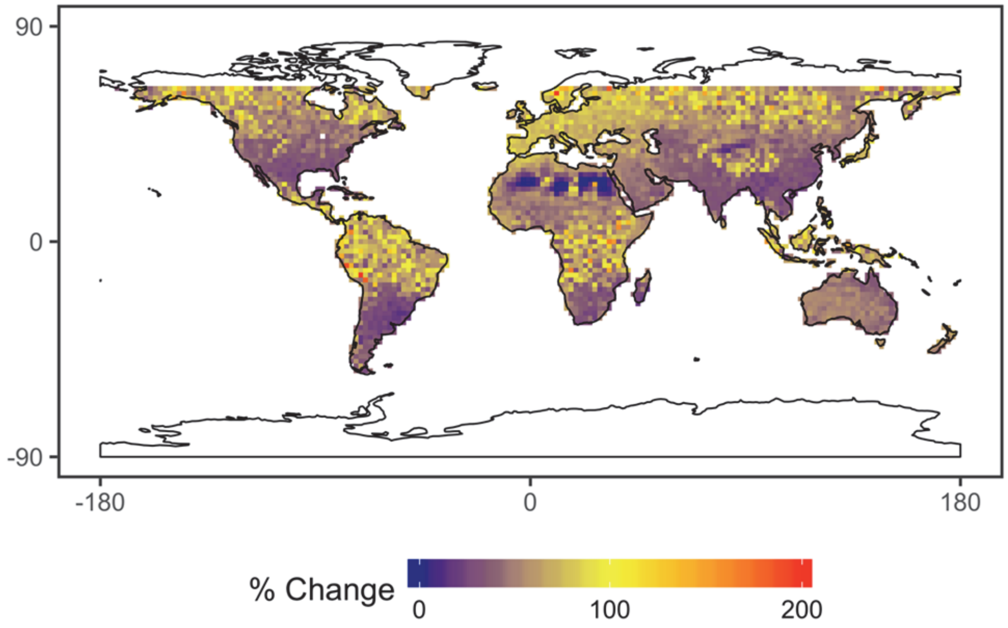

Supplementary Figure 9. Global percent change in heterotrophic biomass for the modern and future worlds. Top figure: percent of Modern world compared to Pleistocene world. Bottom figure: percent of Future world compared to Pleistocene world. The percent change is biomass is lowest in savanna/grassland/desert environments and more in forested (tropical and boreal) regions. Global map from the 110m land polygon shapefile from Natural Earth Data (https://www.naturalearthdata.com/downloads/110m-physical-vectors/).

Supplementary References

1. West, G., Brown, J. H. & Enquist, B. J. A general model for the origin of allometric scaling laws in biology. *Science* **276**, 122–126 (1997).
2. West, G. B., Brown, J. H. & Enquist, B. J. The fourth dimension of life: fractal geometry and allometric scaling of organisms. *Science* **284**, 1677–1679 (1999).
3. West, G., Brown, J. & Enquist, B. A general model for ontogenetic growth. *Nature* (2001).
4. Enquist, B. J., West, G. B., Charnov, E. L. & Brown, J. H. Allometric scaling of production and life-history variation in vascular plants. *Nature* **401**, 907–911 (1999).
5. Enquist, B. J. *et al.* A general integrative model for scaling plant growth, carbon flux, and functional trait spectra. *Nature* **449**, 218–222 (2007).
6. Allen, A. P. & Gillooly, J. F. Towards an integration of ecological stoichiometry and the metabolic theory of ecology to better understand nutrient cycling. *Ecology Letters* **12**, 369–384 (2009).
7. Elser, J. J. J. *et al.* Biological stoichiometry of plant production: metabolism, scaling, and ecosystem response to global change. *New Phytologist* **186**, 593–608 (2010).
8. Strauss, R. E. The study of allometry since Huxley. in 47–75 (Johns Hopkins University Press, 1993).
9. Peters, R. H. *The ecological implications of body size*. *Cambridge studies in ecology* **Cambridge**, (1983).
10. Enquist, B. J., West, G. B. & Brown, J. H. Extensions and evaluations of a general quantitative theory of forest structure and dynamics. *Proceedings of the National Academy of Sciences of the United States of America* **106**, 7046–51 (2009).
11. Enquist, B. J. B. J. & Bentley, L. P. L. P. Land plants: New theoretical directions and empirical prospects. in *Metabolic Ecology: A Scaling Approach* 164–187 (2012). doi:10.1002/9781119968535
12. West, G. B., Brown, J. H. & Enquist, B. J. A general model for the structure and allometry of plant vascular systems. *Nature* **400**, 664–667 (1999).
13. Savage, V. M. M. *et al.* Hydraulic trade-offs and space filling enable better predictions of vascular structure and function in plants. *Proceedings of the National Academy of Sciences of the United States of America* **107**, 22722–22727 (2010).
14. Gillooly, J. F., Charnov, E. L., West, G. B., Savage, V. M. & Brown, J. H. *Effects of size and temperature on developmental time*. *Nature* **417**, 70–73 (2002).
15. Gillooly, J. F. *et al.* The metabolic basis of whole-organism RNA and phosphorus content. *Proceedings of the National Academy of Sciences* **102**, 11923–11927 (2005).
16. Damuth, J. *et al.* Effects of body size and temperature on population growth. *Am. Nat* **163**, 429–441 (2004).
17. Enquist, B. J., West, G. B., Brown, J. H., Enquist, B. J. & Brown, J. H. A general quantitative theory of forest structure and dynamics. *Proceedings of the National Academy of Sciences of the United States of America* **106**, 7040–5 (2009).
18. Savage, V. *et al.* The predominance of quarter-power scaling in biology. *Funct. Ecol* **18**, (2004).
19. Kleiber, M. Body size and metabolism. *Hilgardia: A Journal of Agricultural Science* **6**, 315–353 (1932).

20. Calder, W. A. & Calderiii, W. An allometric approach to population cycles in mammals. *Journal of Theoretical Biology,* **100**, 275–282 (1983).
21. Speakman, J. R. Body size, energy metabolism and lifespan. *The Journal of Experimental Biology* **208**, 1717–1730 (2005).
22. Damuth, J. *et al.* Scaling and power-laws in ecological systems. *Journal of Experimental Biology* **208**, 1749–1769 (2005).
23. Marquet, P. A. and Taper, M. L. On size and area: patterns of mammalian body size extremes across landmasses. *Evol. Ecol.* **12**, 127–139 (1998).
24. Brown, J. H. *Macroecology*. *Macroecology* (1995).
25. Kelt, D. A. & Van Vuren, D. H. The Ecology and Macroecology of Mammalian Home Range Area. *The American Naturalist* **157**, 637–645 (2001).
26. Kelt, D. A. & Van Vuren, D. Energetic constraints and the relationship between body size and home range area in mammals. *Ecology* **80**, 337–340 (1999).
27. Jetz, W., Carbone, C., Fulford, J. & Brown, J. H. The scaling of animal space use. *Science* **306**, 266–268 (2004).
28. Brown, J. H., Marquet, P. A. & Taper, M. L. Evolution of body size: consequences of an energetic definition of fitness. *American Naturalist* **142**, 573–584 (1993).
29. Niklas, K. J. Maximum plant height and the biophysical factors that limit it. *Tree physiology* **27**, 433–40 (2007).
30. Koch, G. W., Sillett, S. C., Jennings, G. M. & Davis, S. D. The limits to tree height. *Nature* **428**, 851–854 (2004).
31. McDowell, N. G. & Allen, C. D. Darcy's law predicts widespread forest mortality under climate warming. *Nature Climate Change* **5**, 669–672 (2015).
32. Ryan, M. G. & Yoder, B. J. Hydraulic Limits to Tree Height and Tree Growth. *BioScience* **47**, 235–242 (1997).
33. Bennett, A. C., Mcdowell, N. G., Allen, C. D. & Anderson-Teixeira, K. J. Larger trees suffer most during drought in forests worldwide. *Nature Plants* **1**, 5139 (2015).
34. Ryan, M. G., Phillips, N. & Bond, B. J. The hydraulic limitation hypothesis revisited. *Plant, Cell and Environment* **29**, 367–381 (2006).
35. Kempes, C. P., West, G. B., Crowell, K. & Girvan, M. Predicting maximum tree heights and other traits from allometric scaling and resource limitations. *PloS one* **6**, e20551 (2011).
36. McDowell, N. G. *et al.* Predicting Chronic Climate-Driven Disturbances and Their Mitigation. *Trends in Ecology and Evolution* **33**, 15–27 (2018).
37. Portner, H.-O. Oxygen- and capacity-limitation of thermal tolerance: a matrix for integrating climate-related stressor effects in marine ecosystems. *Journal of Experimental Biology* **213**, 881–893 (2010).
38. Cheung, W. W. L. *et al.* Shrinking of fishes exacerbates impacts of global ocean changes on marine ecosystems. *Nature Climate Change* **3**, 254–258 (2013).
39. Von Bertalanffy, L. *Theoretische Biologie—Zweiter Band: Stoffwechsel, Wachstum (A. Francke).* (1951).
40. Calderiii, W. An allometric approach to population cycles of mammals. *Journal of Theoretical Biology* **100**, 275–282 (1983).
41. Calder, W. A. Diversity and Convergence: Scaling for Conservation. in *Scaling in Biology* (eds. Brown, J. H. & West, G. B.) 297–323 (Oxford University Press, 2000).
42. Marquet, P. a., Fernández, M., Navarrete, S. a. & Valdovinos, C. Diversity emerging:

towards a deconstruction of biodiversity patterns. *Frontiers of Biogeography: New direction in the Geography nature.* 191–209 (2004).
43. Enquist, B. J., Brown, J. H. & West, G. B. Allometric scaling of plant energetics and population density. *Nature* **395**, 163–165 (1998).
44. White, E. P., Ernest, S. K. K. M. K. M., Kerkhoff, A. J. & Enquist, B. J. Relationships between body size and abundance in ecology. *Trends in ecology & evolution (Personal edition)* **22**, 323–330 (2007).
45. Ernest, S. K. M. *et al.* Thermodynamic and metabolic effects on the scaling of production and population energy use. *Ecology Letters* **6**, 990–995 (2003).
46. MacArthur, R. H. & Wilson, E. O. *The theory of island biogeography / by Robert H. MacArthur and Edward O. Wilson. Monographs in population biology: 1* (1967).
47. Soulé, M. E. & Mills, L. S. No need to isolate genetics. *Science* **282**, 1658–1659 (1998).
48. Tomiya, S. Body size and extinction risk in terrestrial mammals above the species level. *The American Naturalist* **182**, E196-214 (2013).
49. Cardillo, M. *et al.* Evolution: Multiple causes of high extinction risk in large mammal species. *Science* **309**, 1239–1241 (2005).
50. Smith, F., Smith, R. E. E., Lyons, S. K. & Payne, J. L. Body size downgrading of mammals over the late Quaternary. *, Science* **360**, 310–313 (2018).
51. Kelt, D. A. & Brown, J. H. Diversification of body sizes: Patterns and processes in the assembly of terrestrial mammal faunas. *Biodiversity dynamics. Turnover of populations, taxa, and communities* 109–131 (1998).
52. Cardillo, M. *et al.* Evolution: Multiple causes of high extinction risk in large mammal species. *Science* **309**, 1239–1241 (2005).
53. Ripple, W. J. *et al.* Extinction risk is most acute for the world's largest and smallest vertebrates. *Proceedings of the National Academy of Sciences of the United States of America* **114**, 10678–10683 (2017).
54. Enquist, B. J., Michaletz, S. T. & Kerkhoff, A. J. Toward a General Scaling Theory for Linking Traits, Stoichiometry, and Body Size to Ecosystem Function. in *A Biogeoscience Approach to Ecosystems* (eds. Johnson, E. A. & Martin, Y. E.) 9–46 (Cambridge University Press, 2016). doi:10.1017/cbo9781107110632.004
55. West, G. B., Enquist, B. J. & Brown, J. H. Supporting Information. *Proceedings of the National Academy of Sciences* 1–5 (2009).
56. Stegen, J. C. J. C. *et al.* Variation in above-ground forest biomass across broad climatic gradients. *Global Ecology and Biogeography* **20**, 744–754 (2011).
57. Phillips, O. & Miller, J. S. *Global patterns of plant diversity: Alwyn H. Gentry's forest transect data set.* **89**, (Missouri Botanical Press, 2002).
58. Michaletz, S. T. S. T., Cheng, D., Kerkhoff, A. J. A. J. & Enquist, B. J. B. J. Convergence of terrestrial plant production across global climate gradients. *Nature* **512**, 39–43 (2014).
59. Michaletz, S. T., Kerkhoff, A. J. & Enquist, B. J. Drivers of terrestrial plant production across broad geographical gradients. *Global Ecology and Biogeography* **27**, 166–174 (2018).
60. Enquist, B. J. *et al.* Scaling from traits to ecosystems: Developing a general Trait Driver Theory via integrating trait-based and metabolic scaling theories. *Advances in Ecological Research* **52**, 249–318 (2015).
61. Enquist, B. J. *et al.* Assessing trait-based scaling theory in tropical forests spanning a broad temperature gradient. *Global Ecology and Biogeography* **26**, 1357–1373 (2017).

62. Šímová, I. *et al.* The relationship of woody plant size and leaf nutrient content to large-scale productivity for forests across the Americas. *Journal of Ecology* (2019). doi:10.1111/1365-2745.13163
63. Stephenson, N. L. *et al.* Rate of tree carbon accumulation increases continuously with tree size. *Nature* **507**, 90–93 (2014).
64. Michaletz, S. T., Cheng, D., Kerkhoff, A. J. & Enquist, B. J. Convergence of terrestrial plant production across global climate gradients. *Nature* **512**, (2014).
65. Brown, J. H. & Maurer, B. a. Macroecology: the division of food and space among species on continents. *Science (New York, N.Y.)* **243**, 1145–1150 (1989).
66. Doughty, C. E., Wolf, A. & Malhi, Y. The legacy of the Pleistocene megafauna extinctions on nutrient availability in Amazonia. *Nature Geoscience* **6**, 761–764 (2013).
67. Borggrefe, T., Davis, R., Erdjument-Bromage, H., Tempst, P. & Kornberg, R. D. *A complex of the Srb8, -9, -10, and -11 transcriptional regulatory proteins from yeast. Journal of Biological Chemistry* **277**, (2002).
68. Fan, Z. X., Cao, K. F. & Becker, P. Axial and radial variations in xylem anatomy of angiosperm and conifer trees in Yunnan, China. *IAWA Journal* **30**, 1–13 (2009).
69. Riutta, T. *et al.* Logging disturbance shifts net primary productivity and its allocation in Bornean tropical forests. *Global Change Biology* (2018). doi:10.1111/gcb.14068
70. Owen-Smith, R. N. *Megaherbivores: The influence of very large body size on ecology*. (Cambridge University Press, 1988).
71. Estes, J. A. *et al.* Trophic Downgrading of Planet Earth. *Science* **333**, 301–306 (2011).
72. Janzen, D. H. & Martin, P. S. Neotropical anachronisms: the fruits the gomphotheres ate. *Science* **215**, 19–27 (1982).
73. Pires, M. M., Guimarães, P. R., Galetti, M. & Jordano, P. Pleistocene megafaunal extinctions and the functional loss of long-distance seed-dispersal services. *Ecography* (2017). doi:10.1111/ecog.03163
74. Zimov, S. A. *et al.* Steppe-Tundra Transition: A Herbivore-Driven Biome Shift at the End of the Pleistocene. *The American Naturalist* **146**, 765–794 (1995).
75. Sobral, M. *et al.* Mammal diversity influences the carbon cycle through trophic interactions in the Amazon. *Nature Ecology and Evolution* **1**, 1670–1676 (2017).
76. Campos-Arceiz, A. & Blake, S. Megagardeners of the forest - the role of elephants in seed dispersal. *Acta Oecologica* **37**, 542–553 (2011).
77. Blake, S., Deem, S. L., Mossimbo, E., Maisels, F. & Walsh, P. Forest elephants: Tree planters of the congop. *Biotropica* **41**, 459–468 (2009).
78. Bueno, R. S. *et al.* Functional Redundancy and Complementarities of Seed Dispersal by the Last Neotropical Megafrugivores. *PLoS ONE* **8**, (2013).
79. Beschta, R. L. & Ripple, W. J. Large predators and trophic cascades in terrestrial ecosystems of the western United States. *Biological Conservation* **142**, 2401–2414 (2009).
80. McLaren, B. E. & Peterson, R. O. Wolves, Moose, and Tree Rings on Isle Royale. *Science* **266**, 1555–1558 (1994).
81. Doughty, C. E. C. E. *et al.* Megafauna extinction, tree species range reduction, and carbon storage in Amazonian forests. *Ecography* **39**, 194–203 (2016).
82. Hairston, N. G., Smith, F. E. & Slobodkin, L. B. Community structure, population control, and competition. *Am. Nat* **94**, 421–425 (1960).
83. Fretwell, S. D. Food Chain Dynamics: The Central Theory of Ecology? *Oikos* **50**, 291 (1987).

84. Asner, G. P. *et al.* Large-scale impacts of herbivores on the structural diversity of African savannas. *Proceedings of the National Academy of Sciences* **106**, 4947–4952 (2009).
85. Doughty, C. E. *et al.* Megafauna extinction, tree species range reduction, and carbon storage in Amazonian forests. *Ecography* **39**, (2016).
86. Muller-Landau, H. C. Predicting the long-term effects of hunting on plant species composition and diversity in tropical forests. *Biotropica* **39**, 372–384 (2007).
87. Paine, R. T. Food Webs: Linkage, Interaction Strength and Community Infrastructure. *The Journal of Animal Ecology* **49**, 666 (1980).
88. Ostfeld, R. S. & Holt, R. D. Are predators good for your health? Evaluating evidence for top-down regulation of zoonotic disease reservoirs. *Frontiers in Ecology and the Environment* **2**, 13–20 (2004).
89. Young, H. S. *et al.* Declines in large wildlife increase landscape-level prevalence of rodent-borne disease in Africa. *Proceedings of the National Academy of Sciences* **111**, 7036–7041 (2014).
90. Dobson, A. Population dynamics of pathogens with multiple host species. *American Naturalist* **164**, (2004).
91. Cable, J. M., Enquist, B. J. & Moses, M. E. The allometry of host-pathogen interactions. *PloS one* **2**, e1130 (2007).
92. De Leo, G. A. & Dobson, A. P. Allometry and simple epidemic models for microparasites. *Nature* **379**, 720–722 (1996).
93. Althaus, C. L. Of mice, macaques and men: Scaling of virus dynamics and immune responses. *Frontiers in Microbiology* **6**, (2015).
94. Banerjee, S., Perelson, A. S. & Moses, M. Modelling the effects of phylogeny and body size on within-host pathogen replication and immune response. *Journal of the Royal Society, Interface* **14**, 20170479 (2017).
95. Molnár, P. K., Kutz, S. J., Hoar, B. M. & Dobson, A. P. Metabolic approaches to understanding climate change impacts on seasonal host-macroparasite dynamics. *Ecology Letters* **16**, 9–21 (2013).
96. Keesing, F. *et al.* Impacts of biodiversity on the emergence and transmission of infectious diseases. *Nature* **468**, 647–652 (2010).
97. Duncanson, L. I. L. I., Dubayah, R. O. R. O. & Enquist, B. J. B. J. Assessing the general patterns of forest structure: Quantifying tree and forest allometric scaling relationships in the United States. *Global Ecology and Biogeography* **24**, 1465–1475 (2015).
98. Purves, D. *et al.* Time to model all life on Earth. *Nature* **493**, 295–297 (2013).
99. Harfoot, M. B. J. *et al.* Emergent Global Patterns of Ecosystem Structure and Function from a Mechanistic General Ecosystem Model. *PLoS Biology* **12**, (2014).
100. Smith, M. J. *et al.* The climate dependence of the terrestrial carbon cycle, including parameter and structural uncertainties. *Biogeosciences Discussions* **9**, 13439–13496 (2013).
101. Bartlett, L. J., Newbold, T., Purves, D. W., Tittensor, D. P. & Harfoot, M. B. J. Synergistic impacts of habitat loss and fragmentation on model ecosystems. *Proceedings of the Royal Society B: Biological Sciences* (2016). doi:10.1098/rspb.2016.1027
102. McCauley, D. J. *et al.* Marine defaunation: Animal loss in the global ocean. *Science* **347**, (2015).
103. Faurby, S. & Svenning, J. C. Historic and prehistoric human-driven extinctions have reshaped global mammal diversity patterns. *Diversity and Distributions* **21**, 1155–1166

(2015).
104. Gillooly, J. F., Brown, J. H., West, G. B., Savage, V. M. & Charnov, E. L. Effects of size and temperature on metabolic rate. *Science* **293**, 2248–2251 (2001).
105. Nagy, K. A., Girard, I. A. & Brown, T. K. ENERGETICS OF FREE-RANGING MAMMALS, REPTILES, AND BIRDS. *Annual Review of Nutrition* (1999). doi:10.1146/annurev.nutr.19.1.247
106. Kearney, M., Shine, R. & Porter, W. P. The potential for behavioral thermoregulation to buffer 'cold-blooded' animals against climate warming. *Proceedings of the National Academy of Sciences* (2009). doi:10.1073/pnas.0808913106
107. Young, R. A. Fat, energy and mammalian survival. *Integrative and Comparative Biology* (1976). doi:10.1093/icb/16.4.699
108. Geiser, F. Reduction of metabolism during hibernation and daily torpor in mammals and birds: temperature effect or physiological inhibition? *Journal of Comparative Physiology B* (1988). doi:10.1007/BF00692726
109. Clarke, A., Rothery, P. & Isaac, N. J. B. B. Scaling of basal metabolic rate with body mass and temperature in mammals. *The Journal of animal ecology* **79**, 610–9 (2010).
110. Wolf, A., Doughty, C. E. & Malhi, Y. Lateral Diffusion of Nutrients by Mammalian Herbivores in Terrestrial Ecosystems. *PLoS ONE* **8**, (2013).
111. Doughty, C. E. *et al.* Global nutrient transport in a world of giants. *Proceedings of the National Academy of Sciences* **113**, 868–873 (2016).
112. Clauss, M., Schwarm, A., Ortmann, S., Streich, W. J. & Hummel, J. A case of non-scaling in mammalian physiology? Body size, digestive capacity, food intake, and ingesta passage in mammalian herbivores. *Comparative Biochemistry and Physiology - A Molecular and Integrative Physiology* (2007). doi:10.1016/j.cbpa.2007.05.024
113. Blackburn, T. M. & Hawkins, B. A. Bergmann's rule and the mammal fauna of northern North America. *Ecography* (2004). doi:10.1111/j.0906-7590.2004.03999.x
114. Morales-Castilla, I., Rodríguez, M. Á. & Hawkins, B. A. Deep phylogeny, net primary productivity, and global body size gradient in birds. *Biological Journal of the Linnean Society* (2012). doi:10.1111/j.1095-8312.2012.01917.x
115. Sheridan, J. A. & Bickford, D. Shrinking body size as an ecological response to climate change. *Nature Climate Change* **1**, 401–406 (2011).
116. Smith, F. A., Doughty, C. E., Malhi, Y., Svenning, J. C. & Terborgh, J. Megafauna in the Earth system. *Ecography* **39**, 99–108 (2016).
117. Dirzo, R. *et al.* Defaunation in the Anthropocene. *Science* **345**, 401–406 (2014).
118. Svenning, J. C. *et al.* Science for a wilder Anthropocene: Synthesis and future directions for trophic rewilding research. *Proceedings of the National Academy of Sciences of the United States of America* **113**, 898–906 (2016).
119. Sandom, C., Donlan, C. J., Svenning, J. C. & Hansen, D. Rewilding. *Key Topics in Conservation Biology 2* 430–451 (2013). doi:10.1002/9781118520178.ch23
120. Nagy, K. A. Field metabolic rate and body size. *J. Exp. Biol* **208**, 1621–1625 (2005).
