# Supplementary material for: The megabiota are disproportionately important for biosphere functioning
Source: Nat Commun. 2020 Feb 4;11:699. doi: 10.1038/s41467-020-14369-y (PMC7000713; doi:10.1038/s41467-020-14369-y)
Supplement: Supplementary file 3 — Reporting Summary [file 41467_2020_14369_MOESM3_ESM.pdf]

nature research

Corresponding author(s): Brian J. Enquist

Last updated by author(s): Nov 7, 2019

Reporting Summary

Nature Research wishes to improve the reproducibility of the work that we publish. This form provides structure for consistency and transparency in reporting. For further information on Nature Research policies, see Authors & Referees and the Editorial Policy Checklist.

Statistics

For all statistical analyses, confirm that the following items are present in the figure legend, table legend, main text, or Methods section.

n/a	Confirmed	
☐	☒	The exact sample size (*n*) for each experimental group/condition, given as a discrete number and unit of measurement
☐	☒	A statement on whether measurements were taken from distinct samples or whether the same sample was measured repeatedly
☐	☒	The statistical test(s) used AND whether they are one- or two-sided<br>*Only common tests should be described solely by name; describe more complex techniques in the Methods section.*
☐	☒	A description of all covariates tested
☐	☒	A description of any assumptions or corrections, such as tests of normality and adjustment for multiple comparisons
☐	☒	A full description of the statistical parameters including central tendency (e.g. means) or other basic estimates (e.g. regression coefficient) AND variation (e.g. standard deviation) or associated estimates of uncertainty (e.g. confidence intervals)
☐	☒	For null hypothesis testing, the test statistic (e.g. *F*, *t*, *r*) with confidence intervals, effect sizes, degrees of freedom and *P* value noted<br>*Give P values as exact values whenever suitable.*
☐	☒	For Bayesian analysis, information on the choice of priors and Markov chain Monte Carlo settings
☐	☒	For hierarchical and complex designs, identification of the appropriate level for tests and full reporting of outcomes
☐	☒	Estimates of effect sizes (e.g. Cohen's *d*, Pearson's *r*), indicating how they were calculated

Our web collection on statistics for biologists contains articles on many of the points above.

Software and code

Policy information about availability of computer code

Data collection	The source data underlying Figures 4, 5, and Supplementary Figures 2-8; Supplementary Table 2 are provided as a Source Data file The datasets generated during and/or analyzed during the current study are available in the via our github repository https://github.com/andrewjabraham/Megabiota.
Data analysis	The source code underlying the Amazon phosphorus simulation (Fig 4) in Matlab is available via GitHub https://github.com/andrewjabraham/Megabiota. The C# code to run the GEM Madingley simulation is available via GitHub (https://github.com/Madingley/C-sharp-version-of-Madingley). There are good instructions here on how to run the model here should any readers wish to do so. The R codes to analyse the GEM simulations and recreate all associated figures and tables (and Fig 5, Supplementary Figs. 2-8 as well as Table 1 and Supplementary Table 2) are available via GitHub https://github.com/andrewjabraham/Megabiota.

For manuscripts utilizing custom algorithms or software that are central to the research but not yet described in published literature, software must be made available to editors/reviewers. We strongly encourage code deposition in a community repository (e.g. GitHub). See the Nature Research guidelines for submitting code & software for further information.

Data

Policy information about availability of data

All manuscripts must include a data availability statement. This statement should provide the following information, where applicable:

- Accession codes, unique identifiers, or web links for publicly available datasets
- A list of figures that have associated raw data
- A description of any restrictions on data availability

The source data underlying Figures 4, 5, and Supplementary Figures 2-8; Supplementary Table 2 are provided as a Source Data file The datasets generated during and/or analyzed during the current study are available in the via our github repository https://github.com/andrewjabraham/Megabiota.

Field-specific reporting

Please select the one below that is the best fit for your research. If you are not sure, read the appropriate sections before making your selection.

☐ Life sciences ☐ Behavioural & social sciences ☒ Ecological, evolutionary & environmental sciences

For a reference copy of the document with all sections, see nature.com/documents/nr-reporting-summary-flat.pdf

Ecological, evolutionary & environmental sciences study design

All studies must disclose on these points even when the disclosure is negative.

Study description	We extend metabolic scaling theory and use global simulation models to demonstrate that (i) megabiota are more prone to extinction due to human land use, hunting, and climate change; (ii) loss of megabiota has a negative impact on ecosystem metabolism and functioning; and (iii) their reduction has and will continue to significantly decrease biosphere functioning.
Research sample	We provide a theoretical underpinning to: (i) understanding why large animals and trees deserve conservation attention; (ii) the first set of comprehensive predictions for how the loss of the megabiota (the largest plants and animals) will impact (and has impacted) the biosphere; and (iii) policies that emphasize the promotion of large trees and animals on biodiversity, ecosystem processes, and climate mitigation. We first use analytical theory (Metabolic Scaling Theory or MST) to provide a foundation to generate a baseline set of predictions. We show that, in times of global change, the megabiota are more prone to extinction and decreases in their abundances disproportionately influence ecosystem and Earth system processes. We predict that compared to ecosystems where body size ranges are reduced ecosystems with megabiota will disproportionately house more biomass, carbon, and nutrients and will be more fertile. As a result, the loss of large organisms will decrease the metabolism and fertility of the biosphere. These findings also indicate that efforts to conserve both the megabiota and increase the area devoted to conserving the megabiota will have a multiplicative impact on ecosystem functioning. We assessed these predictions within a set global General Ecosystem Model (GEM) set of global simulations. We also test if potential variation in size scaling within complex ecological systems impact variation in ecosystem and biosphere metabolism. Our results underscore the importance of the megabiota to the functioning of the biosphere and to conservation priorities.
Sampling strategy	We utilized global published datasets and a novel simulation model (GEM). This simulation model, the Madingley model General Ecosystem Models or GEM, explicitly simulates the dynamics of plants and all heterotroph organisms between 10 µg and 150,000 kg. The model is mechanistic, generating emergent ecosystem structure and function by simulating a core set of biological and ecological processes at the level of an individual. The complex network of dynamic ecological interactions modelled within the Madingley GEM model can lead to unpredictable behavior. Consequently, to constrain our analysis to a single cause we circumscribed our experiments to manipulating the body size of endothermic herbivores only. For this, we exclusively modelled the terrestrial realm because the megafauna extinctions have been less severe in the oceans than on land
Data collection	We collected simulation data from our GEM approach (see above).
Timing and spatial scale	We used the Madingley Model as it explicitly incorporates the importance of organismal body size (metabolic demands, foraging area, and population dynamics 59; see Supplementary Figure 2). This formulation of a GEM represents complex ecological interaction networks and whole ecosystem dynamics at a global scale58. It is capable of modelling emergent ecosystem and biosphere structure and function by simulating a core set of biological and ecological processes for all terrestrial and marine organisms between 10 µg and 150,000 kg. Details of the simulation model are described in the methods section, supplementary document, supplementary figures 2-9).<br><br>We generated a set of forecasts for how, since the Pleistocene, the downsizing of the terrestrial megafauna has altered or will alter the functioning of ecosystems and biosphere. We ran three sets of simulations, or three different worlds (see Methods and Supplementary Document). In each world, we simulated the loss of the endotherm herbivore megafauna by experimentally changing the maximum attainable body mass. Each world differed in maximum size by an order of magnitude, from 10,000 kg (the largest terrestrial Pleistocene herbivore, Mammuthus columbi), to 1,000 kg (typical modern day maximum size of mammalian taxa) and finally 100 kg (a future world lacking wild megaherbivores). The body mass ranges for all other terrestrial animal cohorts were held constant and approximating those found in the Pleistocene fossil record (Ref58; Table 1; see supplemental Figure 2). We hereafter refer to these three worlds as (i) Pleistocene world, (ii) Modern world; and (iii) Future world.
Data exclusions	There were no data exclusions.
Reproducibility	The source code underlying the Amazon phosphorus simulation (Fig 4) in Matlab is available via GitHub https://github.com/andrewjabraham/Megabiota. The C# code to run the GEM Madingley simulation is available via GitHub (https://github.com/Madingley/C-sharp-version-of-Madingley). There are good instructions here on how to run the model here should any readers wish to do so. The R codes to analyse the GEM simulations and recreate all associated figures and tables (and Fig 5, Supplementary Figs. 2-8 as well as Table 1 and Supplementary Table 2) are available via GitHub https://github.com/andrewjabraham/Megabiota.
Randomization	We used no randomization methods.
Blinding	NA

Did the study involve field work? ☐ Yes ☐ No

Field work, collection and transport

Field conditions	No field work was involved.
Location	NA
Access and import/export	NA
Disturbance	NA

Reporting for specific materials, systems and methods

We require information from authors about some types of materials, experimental systems and methods used in many studies. Here, indicate whether each material, system or method listed is relevant to your study. If you are not sure if a list item applies to your research, read the appropriate section before selecting a response.

Materials & experimental systems

n/a	Involved in the study	
☒	☐	Antibodies
☒	☐	Eukaryotic cell lines
☒	☐	Palaeontology
☒	☐	Animals and other organisms
☒	☐	Human research participants
☒	☐	Clinical data

Methods

n/a	Involved in the study	
☒	☐	ChIP-seq
☒	☐	Flow cytometry
☒	☐	MRI-based neuroimaging
